# Comparative virulence of three different strains of *Burkholderia pseudomallei* in an aerosol non-human primate model

**DOI:** 10.1371/journal.pntd.0009125

**Published:** 2021-02-11

**Authors:** Sylvia R. Trevino, Jennifer L. Dankmeyer, David P. Fetterer, Christopher P. Klimko, Jo Lynne W. Raymond, Alicia M. Moreau, Carl Soffler, David M. Waag, Patricia L. Worsham, Kei Amemiya, Sara I. Ruiz, Christopher K. Cote, Teresa Krakauer

**Affiliations:** 1 Bacteriology Division, United States Army Medical Research Institute of Infectious Diseases, Fort Detrick, Frederick, Maryland, United States of America; 2 Biostatistics Division, United States Army Medical Research Institute of Infectious Diseases, Fort Detrick, Frederick, Maryland, United States of America; 3 Pathology Division, United States Army Medical Research Institute of Infectious Diseases, Fort Detrick, Frederick, Maryland, United States of America; University of Texas Medical Branch, UNITED STATES

## Abstract

Melioidosis, caused by the Gram-negative bacterium *Burkholderia pseudomallei*, is a major cause of sepsis and mortality in endemic regions of Southeast Asia and Northern Australia. *B*. *pseudomallei* is a potential bioterrorism agent due to its high infectivity, especially via inhalation, and its inherent resistance to antimicrobials. There is currently no vaccine for melioidosis and antibiotic treatment can fail due to innate drug resistance, delayed diagnosis and treatment, or insufficient duration of treatment. A well-characterized animal model that mimics human melioidosis is needed for the development of new medical countermeasures. This study first characterized the disease progression of melioidosis in the African green monkey (AGM) and rhesus macaque (RM) for non-human primate model down-selection. All AGMs developed acute lethal disease similar to that described in human acute infection following exposure to aerosolized *B*. *pseudomallei* strain HBPUB10134a. Only 20% of RMs succumbed to acute disease. Disease progression, immune response and pathology of two other strains of *B*. *pseudomallei*, K96243 and MSHR5855, were also compared using AGMs. These three *B*. *pseudomallei* strains represent a highly virulent strain from Thailand (HBPUB101034a), a highly virulent strains from Australia (MSHR5855), and a commonly used laboratory strains originating from Thailand (K96243). Animals were observed for clinical signs of infection and blood samples were analyzed for cytokine responses, blood chemistry and leukocyte changes in order to characterize bacterial infection. AGMs experienced fever after exposure to aerosolized *B*. *pseudomallei* at the onset of acute disease. Inflammation, abscesses and/or pyogranulomas were observed in lung with all three strains of *B*. *pseudomallei*. Inflammation, abscesses and/or pyogranulomas were observed in lymph nodes, spleen, liver and/or kidney with *B*. *pseudomallei*, HBPUB10134a and K96243. Additionally, the Australian strain MSHR5855 induced brain lesions in one AGM similar to clinical cases of melioidosis seen in Australia. Elevated serum levels of IL-1β, IL-1 receptor antagonist, IL-6, MCP-1, G-CSF, HGF, IFNγ, MIG, I-TAC, and MIP-1β at terminal end points can be significantly correlated with non-survivors with *B*. *pseudomallei* infection in AGM. The AGM model represents an acute model of *B*. *pseudomallei* infection for all three strains from two geographical locations and will be useful for efficacy testing of vaccines and therapeutics against melioidosis. In summary, a dysregulated immune response leading to excessive persistent inflammation and inflammatory cell death is the key driver of acute melioidosis. Early intervention in these pathways will be necessary to counter *B*. *pseudomallei* and mitigate the pathological consequences of melioidosis.

## Author summary

Melioidosis, caused by the Gram-negative bacterium *Burkholderia pseudomallei*, is endemic in tropical regions globally and is an emerging threat in non-tropical areas worldwide. Its mortality rate is high in endemic areas due to its high infectivity, antimicrobial resistance, lack of available vaccines and limited treatment options. Animal model development and pathogenicity studies of various isolates are critical for the development of countermeasures against this pathogen. In this study, we compared the virulence of three different isolates of *B*. *pseudomallei* from two geographical locations in an aerosol non-human primate model. We found that early elevations of both pro-inflammatory and anti-inflammatory mediators, as well as the persistence of these mediators in the terminal phase of bacterial infection correlate with mortality. Histopathological analysis showed that the severity of lesions in various organs also correlates with the virulence of the *B*. *pseudomallei* strains, HBPUB10134a, MSHR5855 and K96243. Thus, a dysregulated immune response leading to excessive IL-1β and IL-6 at terminal end points and necrosis are key drivers of acute melioidosis. Development of drugs targeting these host response processes will be necessary to counter *B*. *pseudomallei* and mitigate the pathological consequences of melioidosis. This non-human primate model will facilitate the screening of vaccines and novel therapeutics.

## Introduction

Melioidosis is an opportunistic human infection caused by *Burkholderia pseudomallei*, a saprophytic, gram-negative bacterium. The disease is frequently seen in Southeast Asia and Northern Australia [1–3]. *B*. *pseudomallei* is commonly found in soil, water and rice paddies, in these endemic areas and in tropical regions around the world [4–10]. Melioidosis occurs primarily in humans who have been in contact with contaminated water and soil [2,11]. Inhalation of aerosolized bacteria is also known to be a route of exposure in areas where monsoonal rains, flooding, and high winds occur [12–14]. Acute cases of melioidosis are characterized by fever, malaise, pneumonia, and sepsis [1–3]. Abscess formation can be widespread, and commonly occurs in the liver, spleen, skeletal muscle, prostate, kidney, and occasionally in the brain [11]. The chronic disease of melioidosis is characterized by signs and symptoms that may persist for years [2,11]. The symptoms of chronic melioidosis are usually milder than those of the acute form, making disease diagnosis challenging. The latency period for melioidosis can be extremely long, with infection preceding disease by decades in some cases. Persons exposed to *B*. *pseudomallei* with risk factors such as diabetes, renal failure, and alcoholism are more likely to develop melioidosis [6,11,13,15]. Current treatment consists of combinational antibiotics for prolonged periods and disease relapse is common [16].

*B*. *pseudomallei* is classified as a Tier 1 Select Agent due to the potential for use as a bioweapon [17,18]. There is no vaccine available to prevent disease [19–22] and antibiotic treatment can fail due to inherent resistance of *B*. *pseudomallei* to antibiotics and the persistence of intracellular bacteria [16,21]. The mechanisms of pathogenicity for *B*. *pseudomallei* are not well-characterized, particularly when the disease is acquired by aerosol exposure. Several animal models of melioidosis have been previously developed, most of which involve small rodents, and different routes of exposure, including inhalation [23–32]. Mice injected with *B*. *pseudomallei* develop melioidosis, but the severity of the disease is dependent upon the strain of mice and bacterial isolate used. Irrespective of the route of infection, BALB/c mice are more susceptible to *B*. *pseudomallei* and develop the acute form of disease, whereas C57BL/6 are more resistant and the subsequent disease may be more reflective of a chronic form [23–25,29]. Bacterial colonization is noted in the liver and spleen of various mouse strains and is also characteristic of human disease [22]. However, another rodent model of melioidosis, the Syrian Golden Hamster, is extremely susceptible to infection with *B*. *pseudomallei*, but this extreme sensitivity is not seen in human infection [25,31,32]. A well-characterized animal model of melioidosis that closely mimics human disease is necessary in order to develop medical countermeasures and diagnostic tests for human melioidosis. Non-human primates (NHPs) are phylogenetically related to humans and share many common biological mechanisms, functions and responses to infectious diseases with humans. NHPs also have been successfully used as animal models that mimic human infection for other biothreat agents such as *Yersinia pestis* and *Bacillus anthracis* [33,34]. The goal of this study is to develop an acute model of melioidosis in NHPs for the eventual downstream testing and evaluation of novel medical countermeasures currently being developed. We started with the two species of NHPs, African green monkey (AGM, *Chlorocebus aethiops*) and rhesus macaque (RM, *Macaca mulatta*), for the initial down-selection of an animal model. An early report characterized progression of melioidosis in NHPs exposed to aerosolized *B*. *pseudomallei* strain 1026b, at relatively high doses [35]. Other NHP studies also indicated susceptibilities to aerosolized *B*. *pseudomallei* [36,37] with the common marmoset model showing extreme sensitivity to *B*. *pseudomallei* K96243 [37]. In this study, disease progression and lethality were used to down-select NHPs by using the HBPUB10134a (isolate from Thailand) because based on model development in mice [27,30], this was the most virulent isolate we had in our collection.

Although the virulence of many different isolates of *B*. *pseudomallei* were compared in various murine models of infection [26–30], few comparative studies address the virulence of clinical isolates from diverse geographical locations in NHP models of infection. Isolates from different geographical regions display phylogenetic differences [38] as well as lethality and pathogenicity profiles in animal models of melioidosis [27–30,39]. This study further compares the virulence of three different isolates of *B*. *pseudomallei* from two geographical locations using AGM as the preferred model to recapitulate acute melioidosis. Aerosolized *B*. *pseudomallei* MSHR5855, the most virulent isolate of Australian origin, and K96243, among the most common isolates routinely used in the laboratory, were further characterized in AGM and compared with HBPUB10134a, a hypervirulent strain from Thailand.

## Methods

### Ethics statement

Research was conducted under animal use protocols approved by the USAMRIID Institutional Animal Care and Use Committee (IACUC) in compliance with the Animal Welfare Act, PHS Policy and other Federal statutes and regulations relating to animals and experiments involving animals.

### Bacterial strain selection

A highly virulent strain, HBPUB10134a, obtained from tracheal suction of a patient in Thailand in 2010 [38,40], was used for the first *B*. *pseudomallei* challenge. A side-by-side comparative study was performed to identify the NHP species, AGMs or RMs, as the most suitable model that mimics acute human inhalational melioidosis. By starting with the most virulent strain of *B*. *pseudomallei* (as determined in mice), this provided us with an appropriate benchmark for preliminary model development/evaluation. This benchmark then allowed us to use the minimum number of animals to produce statistically significant observations/data when examining other isolates. Two additional strains, MSHR5855, a geographically distinct strain from a sputum sample collected from a human patient in Australia, in 2011, and K96243, a common laboratory strain obtained from tracheal suction of a septicemic, diabetic female Thai patient in 1993 [38] were used in a second cohort of AGMs (after down-selection to the AGM model was deemed the most appropriate model to be reflective of acute melioidosis). All *B*. *pseudomallei* isolates used in these studies were selected based on association with human disease, virulence in animal models, availability of genetic analysis, and low passage history [38,40].

### Preparation of bacteria

The bacteria were grown in Glycerol Tryptone Broth (GTB), which is composed of 4% glycerol (Sigma Aldrich, St. Louis, MO), 1% tryptone (Difco, Becton Dickinson) and 5% NaCl (Sigma). An overnight (approximately18 h) culture in GTB inoculated from a frozen bacterial stock vial was incubated at 37°C with shaking (200 rpm). The bacteria were then harvested and diluted to the desired concentration based on previous aerosol studies. The actual delivered doses of aerosolized bacteria were then verified by plate counts on sheep’s blood agar (Trypticase soy agar with 5% sheep blood–SBA) plates (ThermoFisher). Inoculated SBA plates were incubated at 37°C for 48 h before enumeration.

### Exposure of NHPs to *B*. *pseudomallei*

Healthy, adult AGMs and RMs of both sexes were obtained from US Army Medical Research Institute of Infectious Diseases (USAMRIID)-approved commercial vendors. The facility where this research was conducted is accredited by the Association for Assessment and Accreditation of Laboratory Animal Care International (AAALACI) and adheres to principles stated in the Guide for the Care and Use of Laboratory Animals, National Research Council, 2011. All animals were at least ≥ 3 kg (and the weights did vary depending upon the species of NHP and sex of the individual animal), seronegative for *B*. *pseudomallei* titers, and free of specific pathogens. Animals were used as close to equal numbers of males and females as possible. Upon receipt, NHPs were given a physical examination and housed with physical enrichment. NHP rooms were maintained on a 12-hour light/dark cycle with temperature and humidity conditions maintained between 64–84°F and between 30–70% humidity. NHPs were fed primate chow daily and supplemented with dietary enrichment (fruits and vegetables) at least 3 times per week. The amount of primate chow given to each NHP was proportional to their weight. NHPs were pair-housed whenever possible, however after infection the NHPs were individually-housed for biosafety/safety reasons. Additional sensory enrichment was provided. For example, in non-biocontainment rooms (pre-infection), NHPs were exposed to radio and television sensory enrichment weekly and NHPs received various forms of manipulanda (e.g. mirrors and toys) throughout the entire study. All NHPs received water through automatic watering systems attached to each cage rack. After exposure to aerosolized *B*. *pseudomallei* early endpoint euthanasia was utilized to limit pain and distress as much as possible. An IACUC approved scoring system was utilized and evaluated clinical characteristics such as (1) appearance (i.e. postural changes); (2) clinical signs (i.e. respiratory rate); (3) natural behavior (i.e. mobility and interactions with peers or staff); (4) provoked behavior (i.e. response to stimuli). Depending upon the score, the outcome was either that the animal remained in normal range, the animal demonstrated significant changes in clinical presentation and warranted checks at least three times per day, or the animal was significantly affected and early endpoint euthanasia was warranted.

Prior to aerosol exposure, AGMs were anesthetized by intramuscular injection with a ketamine/acepromazine mixture (10:1). Telazol was used to anesthetize the RMs. Briefly, NHPs are exposed to aerosolized *B*. *pseudomallei* using a USAMRIID head-only aerosol chamber within a class III biological safety cabinet under the control of an automated bioaerosol exposure system. The target aerosol challenge dose for each animal was calculated from the minute volume determined with a plexiglass whole body plethysmography box performed on the day of exposure to aerosolized bacteria. The total volume of aerosol breathed was determined by the exposure time required to deliver the estimated inhaled dose. The aerosol challenge was generated using a collison nebulizer to produce a highly respirable aerosol (approximately 1–3 micron particles) and samples were collected from an all-glass impinger and analyzed by performing colony forming units (CFU) calculations to determine the inhaled dose of *B*. *pseudomallei*. Five AGMs and five RMs were exposed to an average dose of 351 CFU (range 248–531) of *B*. *pseudomallei* HBPUB10134a. Blood samples were collected from a central vein catheter throughout the in-life phase of the study to include pre- and post-exposure and terminal samples collected at the endpoint for analysis as described below. Endpoint/terminal samples were collected from all NHPs, either euthanized due to clinical conditions or at end of study. VetScan HM5 (Abaxis) was used for complete blood counts (CBCs) of NHPs according to the manufacturer’s instructions. Clinical chemistry analyses were performed with a Piccolo Xpress chemical analyzer (Abaxis) according to manufacturer’s guidelines. For the second cohort, equal numbers of AGMs in each group (n = 5 per group) were aerosolized with an average dose of 206 CFU (range 114–299) of *B*. *pseudomallei* MSHR5855 or 1047 CFU (range 820–1530) of *B*. *pseudomallei* K96243. Due to the fact that K96243 was shown to be less virulent in mouse aerosol models compared to HBPUB10134a and MSHR5855 (in both BALB/c and C57BL/6 mouse strains), a higher target dose was used to ensure clinical disease presentation [30]. Blood samples were obtained pre-exposure (day -7), day 4 PI and prior to euthanasia.

### Telemetry

A radiotelemetry device TA10TA-D70 (Data Sciences International [DSI]) to monitor body temperature was surgical implanted into each NHP in the first cohort at least 21 days prior to exposure. Anesthetics used prior to surgery were identical to those used in aerosol studies described above. Post-operative analgesia was administered at the discretion of the veterinarian and options included buprenorphine (0.01–0.03 mg/kg delivered intramuscularly every 6 to 8 hours), or hydromorphone (0.1–0.2 mg/kg delivered intramuscularly every 6 to 8 hours). Body temperature was recorded every 15 min by the DataQuest A.R.T.4.1 system (DSI). Pre-exposure baseline temperatures were used to calculate a baseline temperature of each NHP. Fever was defined as an elevation of body temperature >1.5°C above baseline values for at least 6 consecutive hours.

### Clinical and activity observations

NHPs were observed at least twice daily for up to 47 days for clinical signs of illness for the first cohort and up to 43 days for the second cohort of NHPs. The scoring parameters were appearance (normal = 0; fixed gaze = 1; abnormal postural changes = 2); respiratory rate (normal 16–40 breaths per min (bpm) = 0; >40 bpm = 1; abdominal breathing = 2); natural behavior (normal/baseline observation = 0; minor changes, less interaction = 1; less mobile and alert or holding onto cage = 2; no interaction, restless, anxious or still = 3); provoked behavior (normal = 0; subdued but responds to stimulation = 1; subdued even to stimulation = 2; unresponsive to gentle prodding = 3). The clinical observation scores are subjective measurements of disease severity based on the above observable parameters, including respiratory function. Early intervention endpoints were used during all studies and NHPs were euthanized when moribund, based on daily clinical observations and in accordance with pre-determined criteria for early-endpoint euthanasia. Predetermined criteria included the following: Scores 0–4 indicated normal NHP; scores of 5–8 required monitoring at least three times per day and analgesia administered (buprenorphine 0.01–0.03 mg/kg given intramuscularly); and scores ≥9 required immediate euthanasia.

### Necropsy and histopathology

A complete necropsy was performed on each animal under BSL-3 containment at the end of the study period or when NHPs were euthanized when early end-point euthanasia criteria was met. For histopathology, all tissues were immersion-fixed in 10% neutral buffered formalin for >21 days. Sections were cut at 5–6 μm on a rotary microtome, mounted on glass slides, and stained with hematoxylin and eosin as previously described [29]. Immunohistochemical (IHC) staining for *B*. *pseudomallei* was performed on all tissues. Serial sections of these tissues were cut and stained for *B*. *pseudomallei* as previously described [30] using a monoclonal mouse antibody to *B*. *pseudomallei* lipopolysaccharide (LPS).

### Tissue homogenate preparation

Portions of lung, spleen, liver, kidney, bronchial lymph nodes and mesenteric lymph nodes were removed and homogenized in PBS 1X using disposable precision homogenizers (Covidien). CFU of the homogenate were determined on SBA with undiluted extract or 10-fold dilutions in sterile PBS (1X). Plates were incubated at 37°C for two to three days before counting CFU.

### Blood and urine preparation

Blood was collected in Wampole isolator 1.5 microbial tubes (Inverness Med. Professional Diag). Sterile collection of urine was done at time of euthanasia. Urine was collected from the bladder using a sterile needled syringe. The urine was then placed in a sterile 50 mL conical container. CFU of the blood or urine were determined on SBA with undiluted sample or 10-fold dilutions in sterile PBS. Plates were incubated at 37°C for two to three days before counting CFU.

### Antibody ELISAs

Immunoglobulin class IgG and IgM titers in challenged NHPs were determined by an ELISA performed in 96-well, Immulon 2 HB, round bottom plates (ThermoFisher). Irradiated *B*. *pseudomallei* K96243 cells were used as antigens and were diluted in 0.1M carbonate buffer, pH 9.5, to a concentration of 10 μg/mL. Diluted cells (50 μL/well) were placed into plates and stored overnight at 4°C. The plates were washed with washing solution (1X PBS, 0.05% Tween 20), and incubated with 100 μL of blocking solution (1X PBS, 1% Casein) for 30 min at 37°C. Two-fold dilutions of NHP irradiated sera were made with antibody assay diluent (1X PBS, 25% Casein) in triplicate, and plates were incubated for 1 h at 37°C. After the plates were washed, 50 μL of 1/5,000-diluted anti-IgG- or anti-IgM-horseradish peroxidase conjugate (Southern Biotechnology Associates) was added to each well, and plates were incubated for 30 min at 37°C. After plates were washed, 50 μL of a buffered hydrogen peroxide and 3, 3´,5, 5´-tetramethylbenzidine solution (Pierce, ThermoFisher) was added to each well, and plates were incubated for 20 min at 37°C. The reaction was stopped with 25 μL of 2N sulfuric acid, and the amount of bound antibody was determined colorimetrically by reading at 450 nm with a reference filter (570 nm). The results are reported as the reciprocal of the highest dilution giving a mean optical density of at least 0.1 (which was at least twice the background) ±1 standard deviation.

### Cytokine/Chemokine expression

Cytokine/Chemokine expression levels in NHP sera were measured by Luminex Mag Pix (Life Technology) as per manufacturer directions. Sera from pre-challenged NHPs were used as normal uninfected controls (baseline). The levels of EGF, eotaxin, FGF-basic, G-CSF, GM-CSF, HGF, I-TAC, IFNγ, IL-10, IL-12, IL-15, IL-17, IL-1β, IL-1RA, IL-2, IL-4, IL-5, IL-6, IL-8, IP-10, MCP-1, MDC, MIF, MIG, MIP-1α, MIP-1β, Rantes, TNFα and VEGF were measured. With the exception of TNFα, only cytokines and chemokines with statistically significant changes from baseline are presented.

### Statistical analysis

Survival times were analyzed by the Kaplan-Meier method, with comparisons between survival curves made by log-rank test. Temperatures collected by radio telemetry were averaged per day, and analyzed by a repeated measures linear mixed effects ANOVA model. Immunoglobulin titers and cytokine concentrations were log transformed prior to analysis and analyzed by a repeated measures linear mixed effects ANOVA model, with results being summarized as the geometric mean (GM), and geometric standard error (GSE) or geometric standard deviation (GSD). CBCs were analyzed analogously, without log transformation, and results summarized as mean and standard error of mean (SEM) or standard deviation (SD). Bacterial counts were analyzed under a negative binomial model, with comparisons between treatment groups made by an approximate *t*-test. In order to adjust for the differing lethality of tested *B*. *pseudomallei* isolates, the association of survival with cytokine concentration was analyzed by a two-way ANOVA (strain x survival) without an interaction term. Analysis was implemented in SAS version 9.4 (SAS Institute Inc., Cary, NC), PROC LIFETEST, PROC GLIMMIX and PROC MIXED. No adjustment was applied for multiple comparisons.

## Results

### Exposure of AGMs and RMs to aerosolized *B*. *pseudomallei* HBPUB10134a

**Fig 1:**
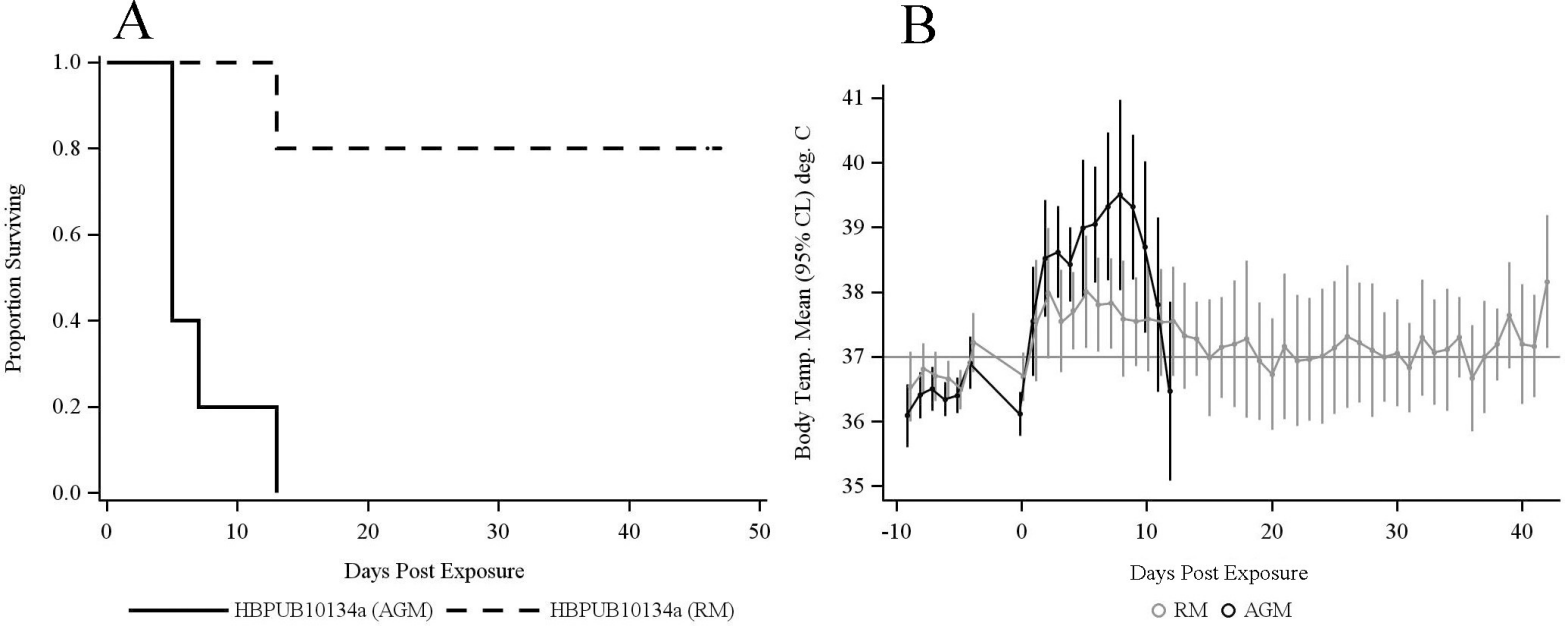
Comparative response of AGMs and RMs challenged with aerosolized *B*. *pseudomallei* HBPUB10134a. Five AGMs and five RMs were exposed to an average dose of 351 CFU (range 248–531) of *B*. *pseudomallei* HBPUB10134a. **(A)** There was a significant difference in survival times between AGMs and RMs by log-rank test (*p* = 0.0041). **(B)** Mean body temperatures were significantly higher in AGMs compared to RMs at early time points. Daily average temperature readings for each animal were computed based on telemetry readings taken at 15 minute intervals. Points and error bars indicate the mean and 95% CL reading for each group (n = 5), and were estimated by a repeated measures linear mixed effects ANOVA model. A difference in the overall time course of the two species was inferred from a statistically significant time by species interaction effect (*p* < 0.0001).

The average exposure dose was 345 CFU (range 248–420 CFU) of *B*. *pseudomallei* HBPUB10134a in the AGM group. This dose resulted in 100% mortality, significantly different from 20% mortality rate observed in RMs infected with 357 CFU (range 261–531 CFU) of HBPUB10134a (Fig 1A, log-rank test *p* = 0.0041). The median time to death in the AGM group was 5 days (95% CL 5.0–13.0). All AGMs and one RM were euthanized due to clinical condition, while four RMs survived to the study endpoint (47 days). All AGMs developed fever (> 1.5°C above basal body temperature) after exposure, with elevation of body temperature persisting for the duration of the study except for one AGM where temperature dropped in the day prior to death (Fig 1B). Four of five RMs developed fever after exposure to HBPUB10134a. Fever developed as early as day 2 PI in AGMs and increases in temperatures were higher than those in RMs at each time point. Increased temperatures were less dramatic in RMs compared to AGMs, with mean temperatures returning to normal by day 14 PI in the RM group. Comparisons between AGMs and RMs indicated a significant overall change in temperature over time between the two groups exposed to HBPUB10134a (time x species interaction, *p* < 0.0001).

**Fig 2:**
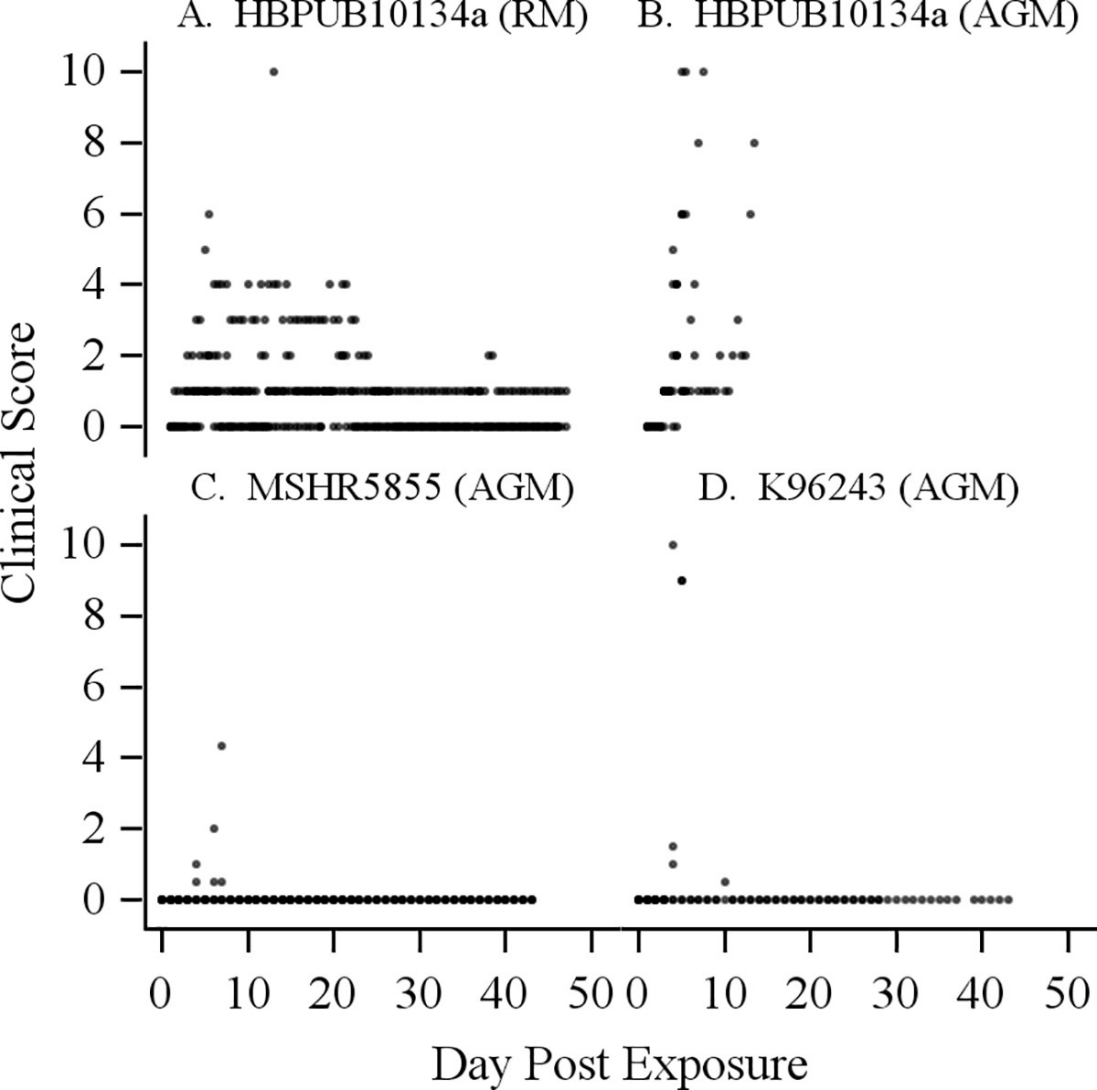
Clinical observation scores of NHPs challenged with aerosolized *B*. *pseudomallei*. (A) RM and (B) AGM response to *B*. *pseudomallei* HBPUB10134a; AGM response to (C) MSHR5855 and (D) K96243. The scoring parameters were appearance (normal = 0; fixed gaze = 1; abnormal postural changes = 2); respiratory rate (normal 16–40 breaths per min (bpm) = 0; >40 bpm = 1; abdominal breathing = 2); natural behavior (normal/baseline observation = 0; minor changes, less interaction = 1; less mobile and alert or holding onto cage = 2; no interaction, restless, anxious or still = 3); provoked behavior (normal = 0; subdued but responds to stimulation = 1; subdued even to stimulation = 2; unresponsive to gentle prodding = 3). The clinical observation scores are subjective measurements of disease severity based on the above observable parameters, including respiratory rates. Each point indicates an animal’s observed clinical score.

Respiratory rate and other clinical signs/activities were also monitored for both NHP groups for the entire length of study. The respiratory rate was slightly higher in AGMs compared to RMs and spikes were seen at terminal time points for the AGM group. RMs also had elevated respiratory rates compared to normal/basal respiration pre-exposure, occurring at multiple time points for the entire observation period but not significantly different from the AGM group. Other clinical signs such as abnormal postural changes, glazed look, altered response to stimulation and provoked activities were also more frequently observed and to a greater extent in AGMs, whereas the four RM survivors had less appreciable changes in clinical signs of disease and activities for the entire observation period (Fig 2A and 2B). All AGMs showed signs of disease with a median clinical score of 1, starting at day 3 PI. Three AGMs had worsening conditions on day 4 PI, with a median score of 4 and were euthanized the next day due to clinical condition (Fig 2B). One AGM had a progressively increasing clinical score over time and was euthanized on day 7 PI. The sole surviving AGM appeared minimally affected up to day 10 PI with a clinical observation score of 1 and a score of 2 on day 12 PI, but then abruptly exhibited severe clinical signs and was euthanized on day 13 PI. Thus, all AGMs exhibited elevated clinical observation scores and were euthanized due to acute signs of disease in response to aerosolized HBPUB10134a.

One RM had signs of disease starting with a clinical observation score of 1 on day 2 PI and the clinical observation score gradually increased to 3 over time between days 3 to 9 PI with a spike of worsening clinical condition on day 5 (Fig 2A). This RM had a fever starting on day 2 and body temperature remained high until day 12 PI. This RM was euthanized the next day due to worsening clinical disease. The rest of the RM group had a median clinical observation score of 1 from days 3 to 5 PI but had no signs of disease from days 7 to 12 PI. However, the condition of one RM fluctuated from days 13 to 24 PI with clinical observation scores of 4 on days 13 to 14 PI and 2 to 3 on subsequent days. This RM then returned to an observation score of 1 from day 25 PI to the end of the study (day 47). The RM group except for one non-survivor was generally less susceptible to HBPUB10134a, the most virulent strain of *B*. *pseudomallei* based on mouse infection models comparing multiple strains of *B*. *pseudomallei* [28,30].

### Exposure of AGMs to aerosolized *B*. *pseudomallei* MSHR5855 or K96243

**Fig 3:**
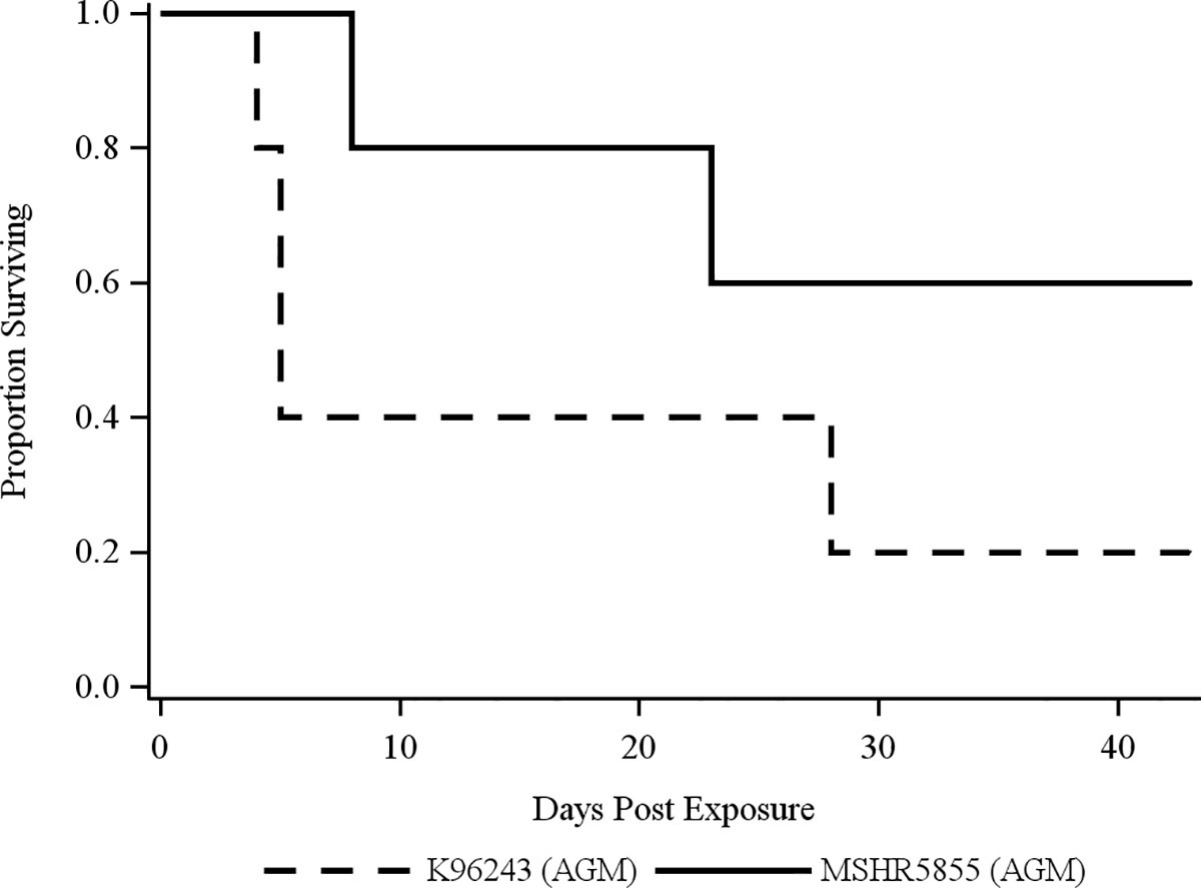
Survival curves of AGMs challenged with aerosolized *B*. *pseudomallei* MSHR5855 and K96243. AGMs were aerosolized with an average dose of 206 CFU (range 114–299) of *B*. *pseudomallei* MSHR5855 or 1047 CFU (range 820–1530) of *B*. *pseudomallei* K96243. There was no significant difference in survival times between these two strains of *B*. *pseudomallei* by log-rank test (*p* = 0.170).

The mechanisms of pathogenicity and virulence among various *B*. *pseudomallei* strains are not well characterized particularly in NHPs, as previous studies focused on *B*. *pseudomallei* 1026b, a less virulent strain originating from a Thai patient in 1993 with unknown passage history [27,40]. Once we established that RM did not meet our objective of developing an acute model for melioidosis, a second cohort of five AGMs in each group was exposed to MSHR5855, a clinical isolate from Australia, or K96243, a commonly used laboratory strain originating from Thailand [38,40]. Fig 3 shows the comparative survival curves of AGMs exposed to either an average aerosol dose of 206 CFU (range 114–299 CFU) of MSHR5855 or 1047 CFU (range 820–1530 CFU) of K96243. Pairwise comparison showed no statistically significant difference of survival rates between these two groups (Fig 3, *p* = 0.17). However, pairwise comparisons with the first cohort of AGMs indicated statistically different survival rates between AGMs exposed to HBPUB10134a and MSHR5855 (*p* = 0.016). It is important to note that the dose of K96243 delivered to the NHP was approximately three times greater than HBPUB10134a and approximately five times greater than MSHR5855. K96243 was used at a higher dose as previous studies in mouse models of infection showed this is a relatively less virulent strain [27,30]. This differential dosing allowed us to begin to characterize the strain differences on disease outcome. Our previous animal data indicated a very low likelihood of clinical manifestations of disease if K96243 was delivered at comparably low doses used for HBPUB10134a and MSHR5855.

The respiratory rate was close to normal for AGMs exposed to MSHR5855, with a slightly higher rate for one AGM on day 28 PI and shallow breathing for another AGM on day 7 PI in this group. The latter AGM appeared to have no signs of disease, with a clinical observation score of 0 up to day 5 PI (Fig 2C). However, the disease in this AGM suddenly worsened on day 7 PI, with an observation score of 4, increasing by 2 points from the previous day.

Pulmonary indicators were more variable among AGMs exposed to K96243. One AGM in the group exposed to K96243 had shallow respiration, labored breathing, and increased respiratory rate on day 4 PI and succumbed to acute disease on the same day. This AGM appeared to have no signs of disease with a clinical observation score of zero from days 1 to 3 PI, but suddenly worsened on day 4 (Fig 2D). Two AGMs in this group had clinical observation scores of 1 and elevated respiratory rates on day 4 PI but otherwise appeared normal from days 1 to 3 PI. Both of these AGMs became severely affected with higher respiratory rates on day 5 PI and were euthanized on the same day due to clinical disease. The remaining two AGMs had no appreciable clinical signs of disease from days 4 to 9 PI, except for labored breathing in one AGM on day 5 and increased respiratory rate on days 24 to 28 PI for the other AGM. The latter AGM appeared to have no signs of disease until day 9 PI and appeared to show marginal signs of disease between days 10 to 28 PI with an average clinical score of 0.5 to 1 based on the two daily observations. However, this AGM had an abdominal soft tissue mass on day 28 PI and was euthanized on the same day due to clinical condition. The remaining survivor in the K96243 group had normal respiratory rate and activity during the entire duration of the study (days 1 to 43).

### Complete blood counts (CBCs) and blood chemistry changes

**Fig 4:**
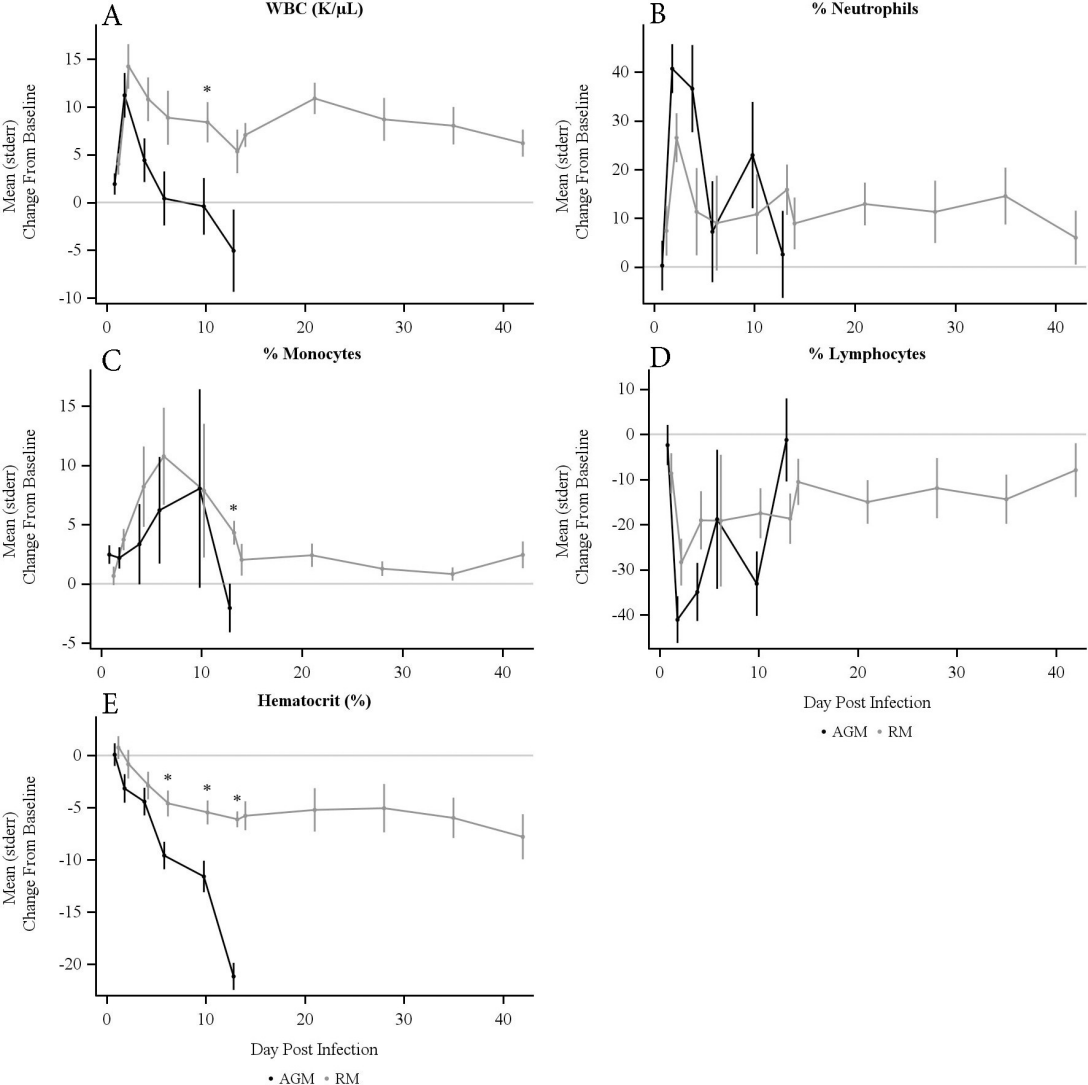
**Changes from baseline in (A) white blood cell counts (WBC), (B) neutrophils, (C) monocytes, (D) lymphocytes, and (E) hematocrit over time in AGMs and RMs exposed to aerosolized *B*. *pseudomallei* HBPUB10134a.** * denotes statistically significant difference between AGM and RM groups by *t*-test (*p* < 0.05). Points indicate mean, with error bars extending to one SEM. Significant differences were found in (A) WBC, (C) % Monocytes, and (E) % Hematocrit from days 7 to 13 PI. Neither (B) % Neutrophils nor (D) % Lymphocytes showed significant differences between the species.

Changes in CBCs and blood chemistry were compared to baseline values obtained prior to exposure to bacteria. Both AGMs and RMs had increases in white blood cell (WBC) counts, peaking on day 2 after exposure to aerosolized HBPUB10134a (Fig 4A). Both AGM and RM groups had neutrophilia peaks on day 2 PI, with higher neutrophilia in AGMs compared to RMs (Fig 4B). Moderate monocytosis and severe lymphopenia were noted for both groups with some variation in time and duration PI (Fig 4C and 4D). Hematocrits for all NHPs trended down over the duration of the study (Fig 4E). Very low hematocrit was observed in AGMs especially at terminal time points and a moderate decrease in hematocrit was measured in RMs over the entire observation period. Hematocrits were significantly different between AGMs and RMs from days 7 to 13 PI (*p* < 0.02).

The CBCs changes observed in AGMs exposed to MSRH5855 or K96243 collected on day 4 PI were not significantly different from that of AGMs exposed to HBPUB10134a. Thus, increased WBC and neutrophils were measured upon infection with all three strains of *B*. *pseudomallei*. Severe lymphopenia was also observed after infection with all three strains of *B*. *pseudomallei*, but hematocrit was lowered only with K96243 and HBPUB10134a infection.

Normal values of asparate transaminase (AST) were observed in both AGMs and RM exposed to HBPUB10134a (Table 1). Significant elevation of AST was seen at terminal time points with AGMs but not in RMs. Higher alkaline phosphatase (ALP) levels were observed in AGMs but not in RMs at day 4 PI. However, ALP levels were elevated in RMs but not in AGMs at terminal time points. No significant changes of lactate dehydrogenase (LDH) were observed in both AGMs and RMs at both time points with HBPUB10134a. However, none of these changes in AST, LDH, and ALP correlated with disease severity or survival in AGMs or RMs exposed to HBPUB10134a. Blood urea nitrogen (BUN) was unchanged in both groups of NHPs at terminal end points. Moderate levels of the acute phase protein, C-reactive protein (CRP), were measured in most NHPs at early time points regardless of the strains of *B*. *pseudomallei* (Table 1). CRP levels were not determined at terminal time points in AGMs exposed to HBPUB10134a.

**Table 1:** Chemistry and other clinical manifestation of inhalation melioidosis.

		HBPUB1034a				K96243		MSHR5855	
		AGM		RM		AGM		AGM	
		n	Mean(SEM)	n	Mean(SEM)	n	Mean(SEM)	n	Mean(SEM)
**AST**	**Baseline**	5	59.4 (4.94)	5	33.2 (2.78)	5	50.2 (3.99)	5	45.4 (6.87)
**(U/L)**	**Day 4**	5	77.4 (10.78)	5	42.8 (9.53)	5	91.2 (16.13)	5	44.4 (6.95)
	**Terminal**	5	250.0 (61.72) *	5	29.8 (6.91)	4	120.3 (58.69)	3	40.0 (5.51)
**ALP**	**Baseline**	5	80.0 (11.45)	5	167.2 (14.33)	5	91.8 (23.14)	5	79.2 (19.46)
**(U/L)**	**Day 4**	5	178.4 (17.01) *	5	244.8 (43.59)	5	129.0 (24.35)	5	97.6 (8.91)
	**Terminal**	5	238.6 (81.06)	5	545.6 (107.27) *	4	334.0 (182.36)	3	113.3 (24.48)
**LDH**	**Baseline**	ND^a^ ND				4	255.8 (33.13)	5	233.4 (7.55)
**(U/L)**	**Day 4**	5	2522.6 (542.95)	5	1838.8 (461.47)	4	710.3 (155.23)	5	256.0 (15.50)
	**Terminal**	3	4684.0 (1231.66)	5	1174.4 (271.74)	3	773.0 (227.00)	3	203.3 (21.11)
**BUN**	**Baseline**	5	18.8 (3.41)	5	13.8 (0.86)	5	16.2 (2.35)	5	15.4 (1.60)
**(mg/dL)**	**Day 4**		ND ND			5	26.8 (10.30)	5	11.8 (2.13)
	**Terminal**	2	64.5 (14.50)	1	36.0 (.)	4	32.8 (12.71)	3	5.7 (0.88) *
**CRP**	**Baseline**	5	8.8 (1.36)	5	4.4 (0.24)	5	5.0 (0.00)	5	5.0 (0.00)
**(mg/L)**	**Day 4**	3	33.4 (12.82)	1	73.0 (.)	5	72.9 (20.85)	5	23.3 (3.91)
	**Terminal**		ND	5	28.5 (13.63)	3	86.3 (30.48)	3	5.0 (0.00)

*indicates significant change from baseline by Welch’s *t*-test (*P* < 0.05).

^a^ND, not determined. Standard reference range for LDH was 402–1364 U/L.

Blood chemistry was normal for most AGMs exposed to MSHR5855 at both day 4 PI and terminal time points except for lower BUN values at terminal end points (Table 1). With K96243 infection, levels of AST, ALP, LDH, BUN, and CRP levels trended insignificantly higher at both time points. Overall, there was no correlation of blood chemistry with disease activity and survival times with *B*. *pseudomallei* infection in NHPs.

### Cytokine/chemokine profiles post-infection

**Fig 5:**
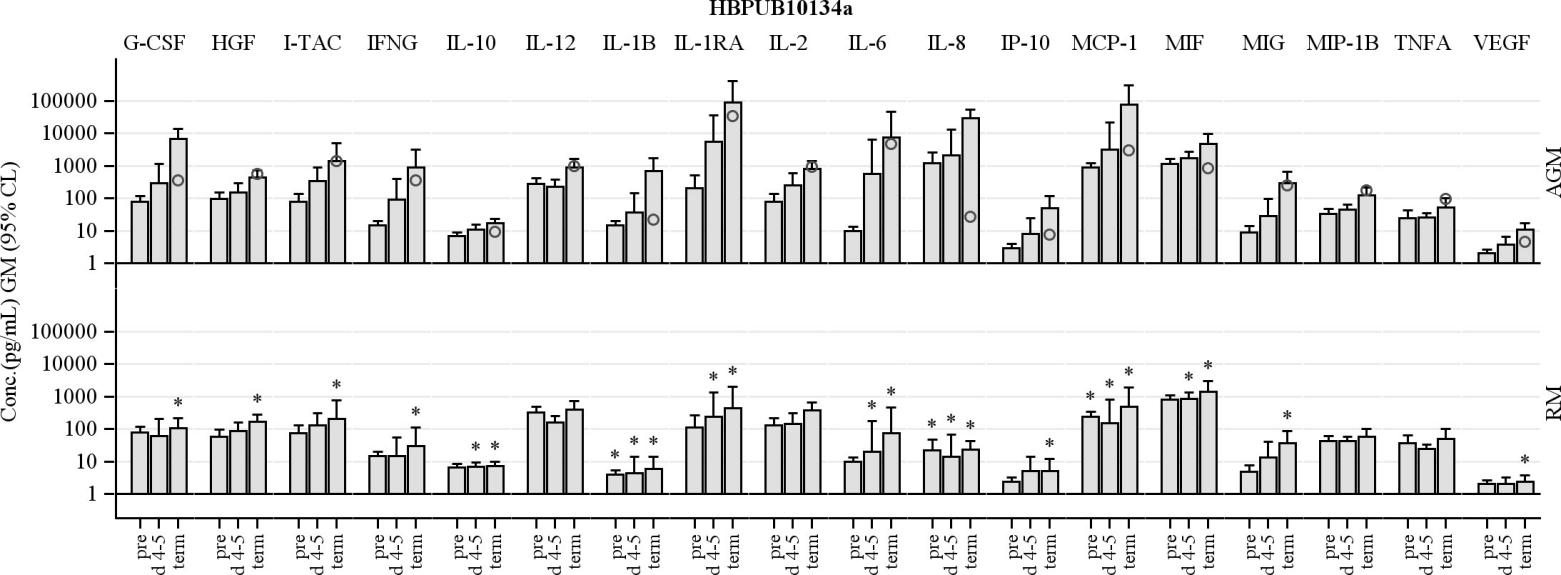
Comparison of serum cytokine and chemokine levels in AGMs and RMs aerosolized with *B*. *pseudomallei* HBPUB10134a. Levels of G-CSF, HGF, I-TAC, IFNG, IL-10, IL-1β, IL-1RA, IL-6, IL-8, IP-10, MCP-1, MIF, MIG and VEGF were measured in sera at baseline, days 4–5 post exposure, and at the terminal bleed. * indicates a statistically significant difference between AGMs and RMs within time point (*p* < 0.05). Plotted values indicate the geometric mean (GM) concentration, with error bars extending to the 95% CL. The terminal time point includes both animals that succumbed, and those that were euthanized at the end of the study. All AGM succumbed to the challenge, while only one RM succumbed. To illustrate the potential effect of succumbing, an open circle is plotted along with the terminal time point of AGM to indicate the concentration of the specific cytokine or chemokine in the moribund RM.

Cytokines and chemokines were assayed in serum samples collected from all NHPs exposed to *B*. *pseudomallei*, with analysis focused on one early time point (day 4/5) and terminal samples. Upon infection with HBPUB10134a, AGMs exhibited 100-fold increase in IL-1 receptor antagonist (IL-1RA) and IL-6 at day 5 PI (Fig 5). Moderate increases in G-CSF, I-TAC, IFNγ, IL-10, IL-1β, IL-2, IL-8, IP-10, MCP-1, MIF, MIG and VEGF were seen at this early time point for AGMs. Terminal levels of all of these mediators were also higher than baseline control blood samples obtained prior to infection in AGMs. In contrast, only small increases (< 1.5-fold) of few cytokines and chemokines were observed in RMs when compared to those of AGMs after HBPUB10134a challenge at both time points. Significantly higher levels of IL-10, IL-1β, IL-1RA, IL-6, IL-8, MCP-1 and MIF were observed in AGM versus RM at day 5 PI (Fig 5, *p* < 0.05). Increases in cytokines and chemokines, G-CSF, HGF, I-TAC, IFNγ, IL-10, IL-1β, IL-1RA, IL-6, IL-8, IP-10, MCP-1, MIF, MIG and VEGF in AGMs were significantly different from RMs at terminal time points post-infection (*p* < 0.05). This cytokine response in AGMs and RMs reveals innate differences between the two NHP species upon infection with *B*. *pseudomallei* HBPUB10134a.

**Fig 6:**
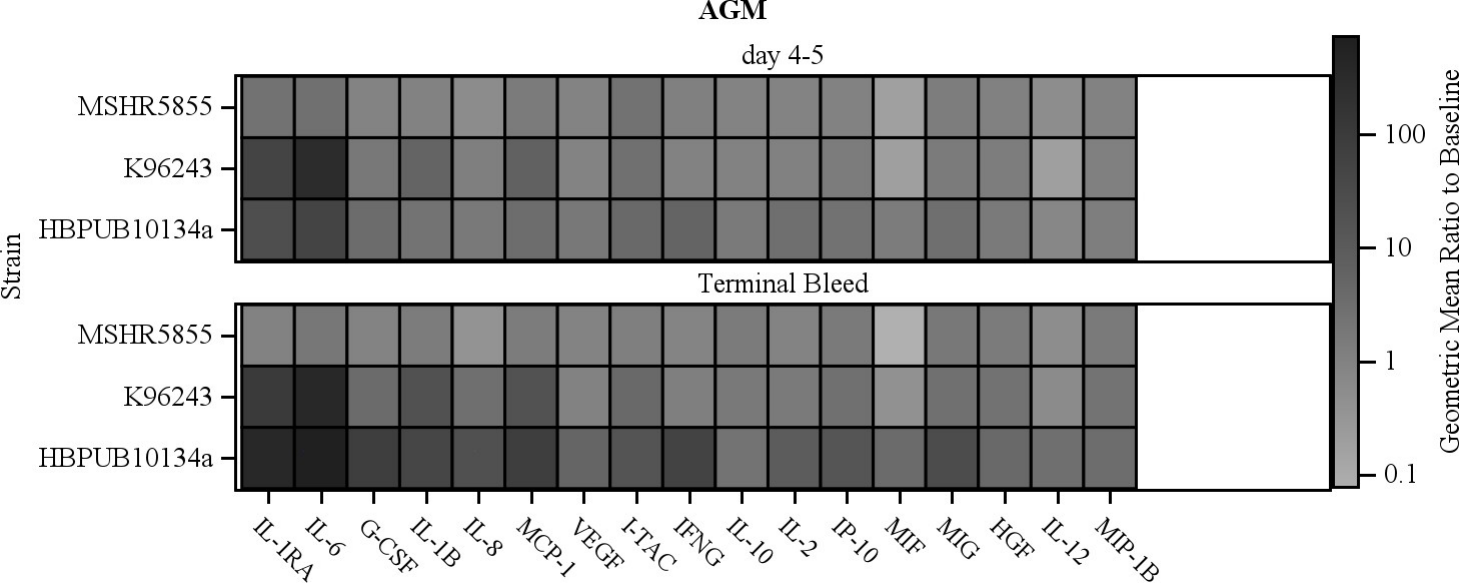
Comparison of increases in cytokine and chemokine levels in AGMs aerosolized with *B*. *pseudomallei* HBPUB10134a, K96243 and MSHR5855. The color scale reflects the geometric mean of the fold increase in serum concentration relative to pre-exposure levels. The increase in these important mediators was generally greater in the more virulent HBPBUB10134a and K96243 strains when compared to MSHR5855.

Similar patterns of elevated cytokines and chemokines were observed in AGMs infected with MSHR5855 and K96243 at early time points with minor exceptions. Fig 6 compares the cytokines and chemokines produced by AGMs in response to these three strains of *B*. *pseudomallei*. HBPUB10134a and K96243 generally induced comparably higher levels of mediators than MSHR5855, with cytokines in terminal blood samples from MSHR5855 returning close to baseline, except for IL-10, IL-6, IP-10, MCP-1 and MIG. Higher levels of IL-6 (> 1000-fold increase) were seen in AGMs infected with K96243 compared to HBPUB10134a-infected AGMs, at days 4–5 PI. IFNγ was induced >10-fold at day 5 PI, increasing to near 100-fold at terminal time point with HBPUB10134a infection, whereas insignificant amounts (< 1.5-fold) were measured in sera of AGMs exposed to either K96243 or MSHR5855. TNFα, a cytokine generally associated with pathogenic bacterial infections in humans [41,42] and experimental animal models of melioidosis [26,28], was notably absent in all NHPs exposed to *B*. *pseudomallei*. VEGF, an important mediator of vascular angiogenesis, was induced only by HBPUB10134a in AGMs (Fig 6).

**Fig 7:**
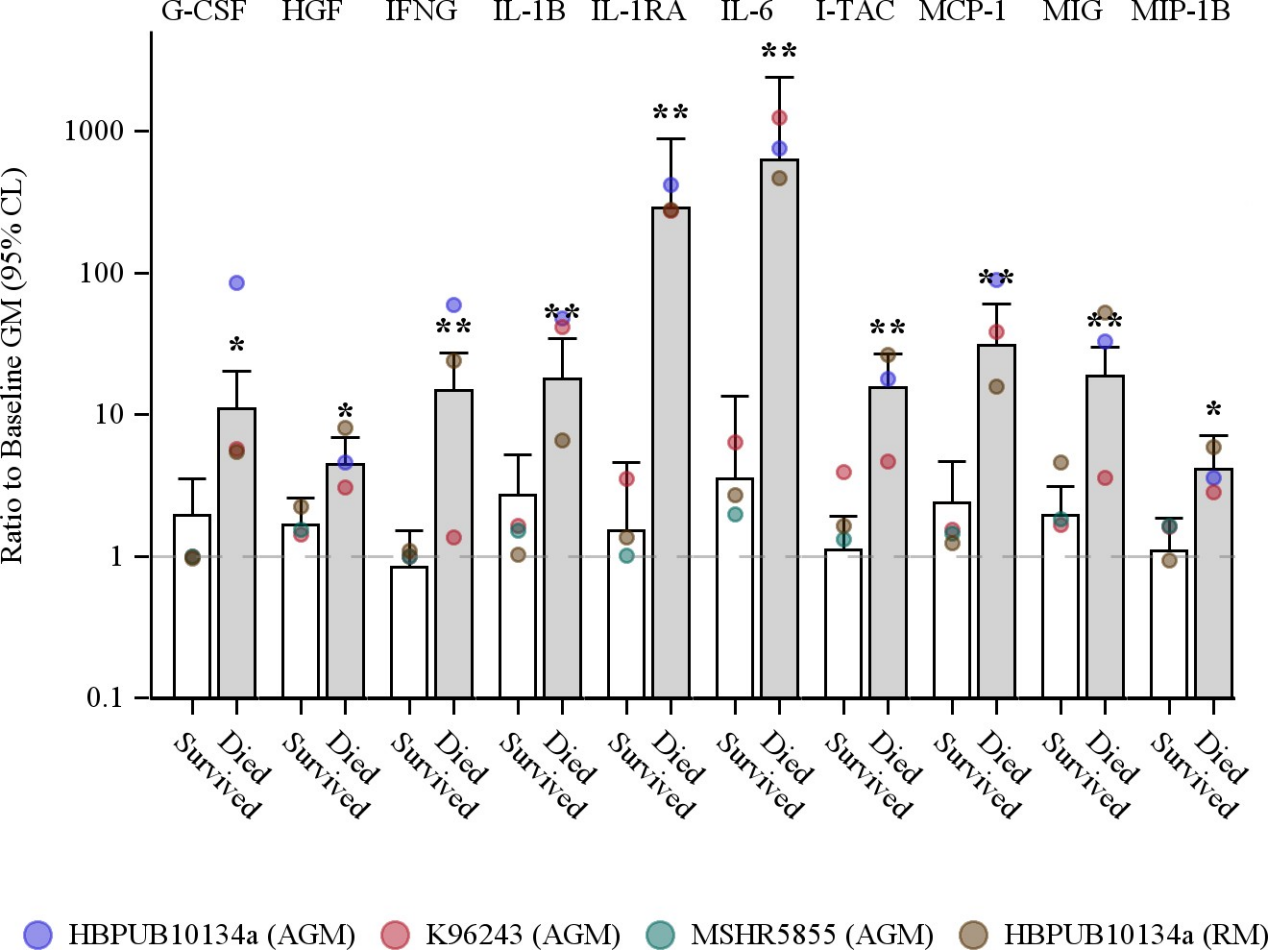
Association of cytokines and chemokines with survival among NHPs aerosolized with *B*. *pseudomallei*. Survival was associated with reduced serum levels of some cytokines and chemokines at the terminal bleed. Colored points indicate the group specific geometric means. Bars are estimates of the geometric mean fold increase in concentration and, to adjust for the differing lethality of the strains, reflect the marginal (least square) means of the 2-way ANOVA model (survival x strain/species) without an interaction term. Note that some groups contain only survivors, or only non-survivors. Error bars indicate the extent of the 95% CL. Statistically significant differences between survivors and non-survivors, based on the main effect contrast of the ANOVA model, are indicated by * (*p* < 0.05) and ** (*p* < 0.01).

As cytokines and chemokines are potent activators of innate immune cells, a statistical analysis of induced cytokines and chemokines to *B*. *pseudomallei* infection outcome was performed. Fig 7 shows that serum levels of G-CSF, HGF, IFNγ, IL-1β, IL-1RA, IL-6, I-TAC, MCP-1, MIG and MIP-1β at the terminal time points were all significantly higher in non-survivors than survivors. Due to the low levels of IL-8 from AGMs infected with MSHR5855 or K96243, IL-8 did not reach a statistically significant correlation with lethality.

### Antibody response

**Fig 8:**
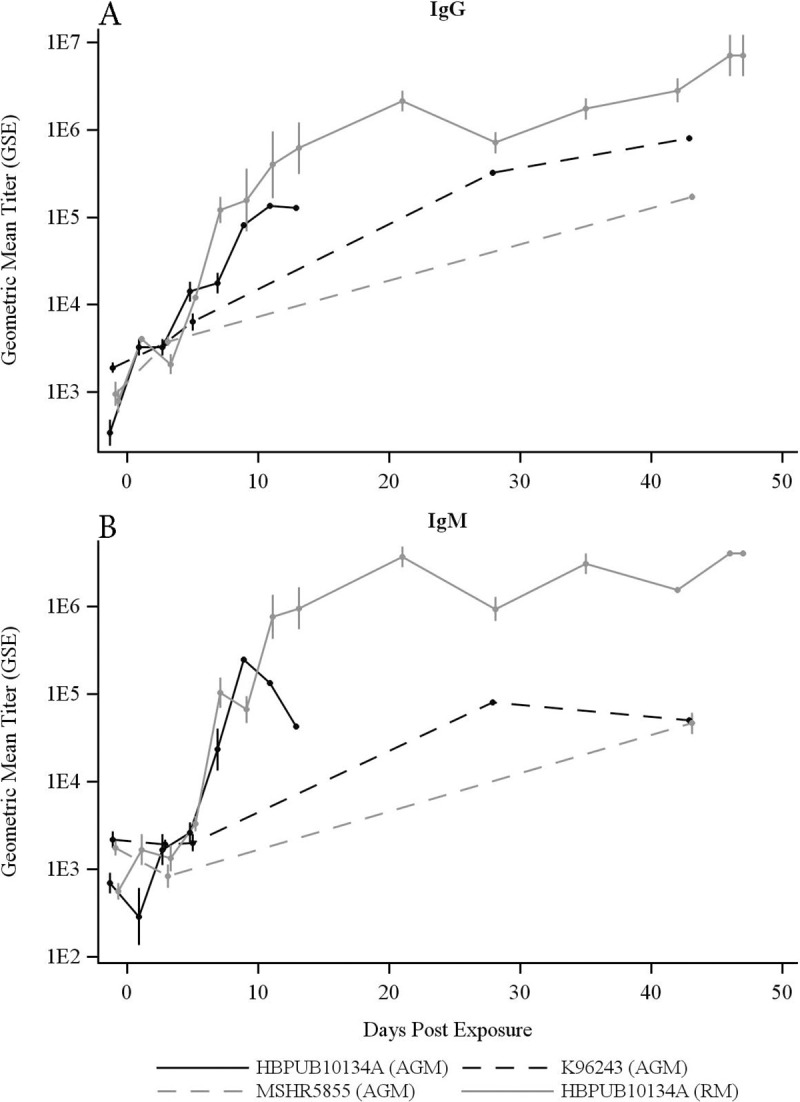
**Serum levels of (A) IgG and (B) IgM in NHPs aerosolized with *B*. *pseudomallei* HBPUB10134a, K96243 and MSHR5855**. Following exposure to HBPUB10134a, the 5 AGM euthanized from day 5 to 13 had terminal GM (GSD) IgG and IgM titers of 20,950(2.67) and 4,314 (2.18), respectively. The 5 AGM exposed to K96243 and euthanized on days 4 to 43 had terminal IgG and IgM titers of 33,355 (12.38) and 7,651 (7.01), respectively, and the 5 AGM exposed to MSHR5855 and euthanized on days 8 to 43 had IgG and IgM titers of 172,355 (1.14) and 46,661 (1.62). By contrast, the 5 RM euthanized from day 13 to 47 had terminal IgG and IgM titers of 4,940,009 (2.60) and 3,964,462 (1.05), respectively. The AGM groups were each significantly different from RMs exposed to HBPUB10134a, for both IgG and IgM, by *t*-test (p < 0.05).

The antibody response of NHPs to *B*. *pseudomallei* was also examined to investigate the humoral response post-infection. The IgG and IgM titers in all NHPs were measured at various time points after exposure to *B*. *pseudomallei*. As most AGMs exposed to HBPUB10134a were euthanized due to the clinical manifestation of infection within the first week after exposure to aerosolized *B*. *pseudomallei*, the IgG levels measured in survivors in the second week of infection were generally lower than serum levels of IgG in RMs at day 14 PI. Serum IgG levels in RMs continued to increase with time until the end of the study, reaching a mean titer of 1 x10^7^ (Fig 8). IgG levels in AGMs measured post-infection with MSHR5855 and K96243 were similar to those post-HBPUB10134a infection at days 3 to 7 with continued increases over time. However, IgG levels at the end of the study period in both MSHR5855- and K96243-exposed AGMs were also lower than HBPUB10134a-exposed RMs. IgM levels of RMs post-HBPUB10134a infection were also comparable to that of IgG at terminal time points. IgM titers of AGMs against all three strains of *B*. *pseudomallei* were significantly lower than those for RMs after HBPUB10134a challenge at terminal time points (*p* < 0.05). Overall, the antibody response against *B*. *pseudomallei* in AGMs was lower than the response from HBPUB10134a-infected RMs.

### Bacteremia

Whole blood samples to determine bacterial counts were collected every day PI for the first week PI and at terminal time points. One AGM was bacteremic from days 1 to 7 PI to HBPUB10134a, but was clear of bacteria at the terminal time point (Table 2). The remaining four AGMs became bacteremic later, between days 3 to 5 PI, and all had positive blood cultures at terminal time points. In the RM group, the RM that had lethal infection was bacteremic starting on day 6 PI and at the terminal time point. One RM that recovered from acute disease based on clinical observations, and survived to the end of the study, was bacteremic only at the study end point. Another RM had a positive blood culture on day 2 PI, but was not bacteremic for the rest of the study period. Thus, four out of five AGMs were bacteremic whereas only two out of five RMs were bacteremic at terminal time points. Bacterial counts were significantly higher in AGMs compared to those from RMs from days 1 to 7 PI after aerosolized HBPUB10134a.

**Table 2:** Bacteremia in AGMs and RMs following inhalation of *B*. *pseudomallei* HBPUB10134a.

	Prevalence		Mean(SE)^c^	
Day Post-Infection	AGM	RM	AGM	RM
1	1 / 5	0 / 5	2.85 (0.62)	0
2	1 / 5	1 / 5	2.85 (0.62)	-0.18 (0.97)
3	2 / 5	0 / 5	2.85 (0.62)^a^	0
4	3 / 5	0 / 5	3.90 (0.62)	0
5	4 / 4	0 / 5	3.52 (0.69)	0
6	1 / 2	1 / 5	4.03 (0.98)	0.30 (1.07)
7	2 / 2	0 / 5	4.31 (0.98)	0
Terminal Bleed	4 / 5	2 / 5^b^	4.04 (0.61)	4.66 (0.59)

^a^One animal with “too numerous to count” values at all dilutions was imputed to 3500 CFU/mL.

^b^Terminal end point for RMs was day 46 or day 47 except for one RM with day 13 as terminal end point. The median time to death in the AGM group was 5 days (95% CL5.0–13.0).

^c^Mean Log_10_ CFU/mL for blood and urine, or Log_10_ CFU/g tissue for other organs. SE represents standard error computed under a negative binomial model.

For K96243 and MSHR4855, blood samples from only four AGMs of each group were available for analysis of bacteria at terminal time points. None of the AGMs exposed to MSHR5855 were bacteremic, whereas two out of four AGMs from the K96243-exposed group were bacteremic (Table 3).

**Table 3:** Bacteremia and bacterial burdens post-exposure to *B*. *pseudomallei*.

	HBPUB10134A				K96243		MSHR5855	
	AGM		RM		AGM		AGM	
	Prev.^a^	Mean(SE)^b^	Prev.	Mean(SE)	Prev.	Mean(SE)	Prev.	Mean(SE)
Blood	4 / 5	4.04 (0.61)	2 / 5	4.66 (0.59)	2 / 4	2.84 (0.66)	0 / 5	--
Lung	5 / 5	6.84 (0.59)	2 / 5	6.50 (0.59)	4 / 5	8.87 (0.59)	3 / 5	3.05 (0.59)
Spleen	5 / 5	6.34 (0.41)	4 / 5	4.98 (0.41)	4 / 5	4.77 (0.41)	2 / 5	1.96 (0.42)
Liver	5 / 5	5.27 (0.47)	2 / 5	4.62 (0.47)	4 / 5	4.93 (0.47)	1 / 5	-0.39 (0.89)
Kidney	5 / 5	3.65 (0.43)	2 / 5	3.00 (0.43)	4 / 5	4.32 (0.43)	1 / 5	0.74 (0.51)
TBLN	5 / 5	5.68 (0.31)	5 / 5	4.58 (0.31)	4 / 5	3.72 (0.31)	2 / 5	2.91 (0.32)
MLN	5 / 5	3.44 (0.33)	3 / 5	2.27 (0.36)	2 / 5	2.36 (0.33)	1 / 5	0.32 (0.63)
Urine	4 / 5	2.92 (0.43)	1 / 4	1.00 (0.53)	2 / 3	3.16 (0.56)	0 / 5	--

^a^ Prevalence (number of NHPs with any bacteria / total number of NHPs)

^b^Mean Log_10_ CFU/mL for blood and urine, or Log_10_ CFU/g tissue for other organs.

SE represents standard error computed under a negative binomial model.

### Bacterial burden in organs

**Fig 9:**
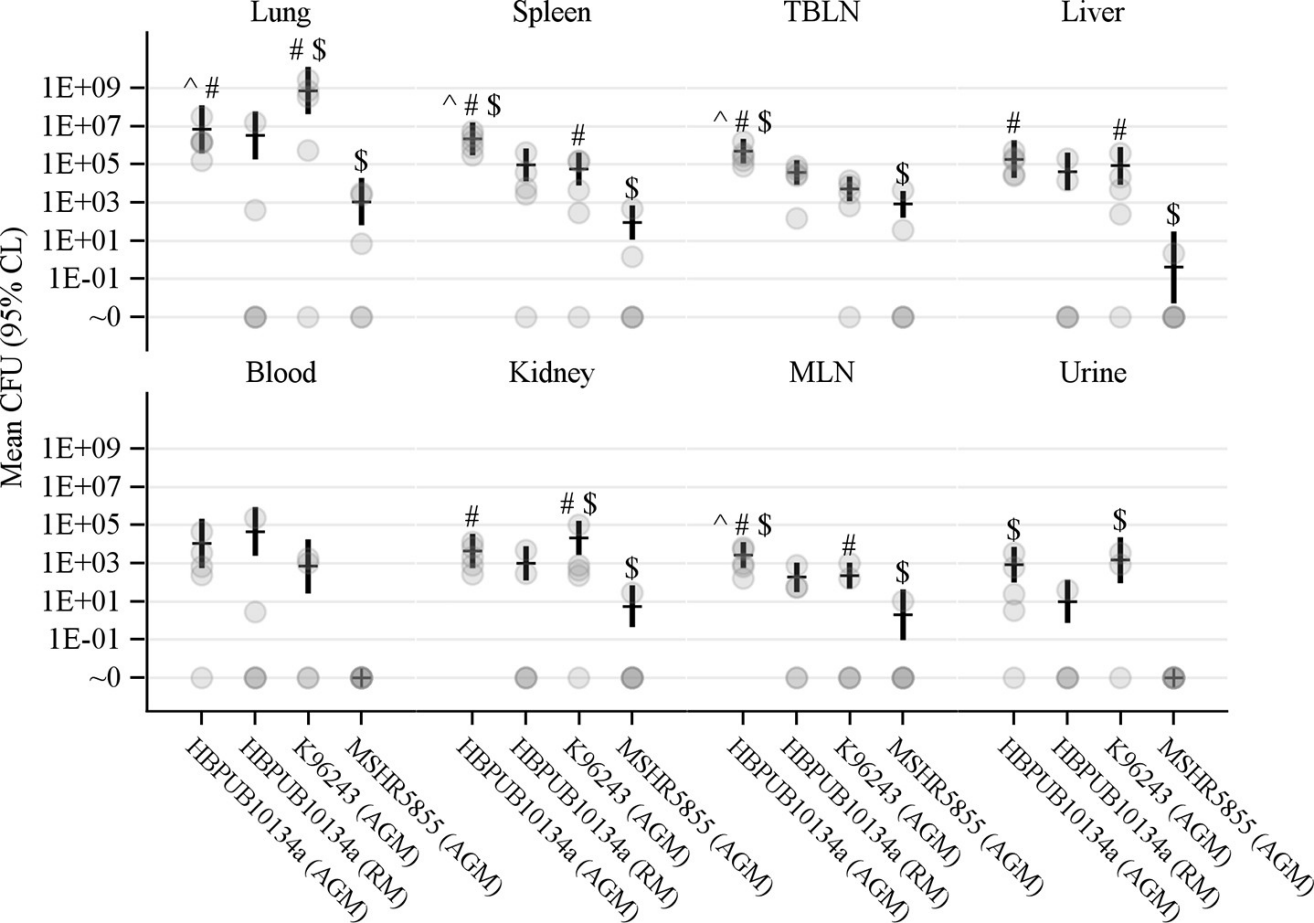
Organ burdens of NHPs aerosolized with *B*. *pseudomallei*. Estimates of the mean CFU count per gram tissue (organs), or per mL (blood and urine) obtained by negative binomial regression, are shown as a dash, with error bars showing the 95% CL. Gray circles indicate the values obtained for each animal. Symbols indicate *p* < 0.05 by an approximate *t*-test: ^ vs AGM/K96243, # vs AGM/MSHR5855, $ vs RM/HBPUB10134a.

At the conclusion of the study, tissue samples were harvested from euthanized NHPs, and processed to determine tissue bacterial burden as previously described [30]. Of all the tissues sampled, lungs contained the highest bacterial loads, irrespective of the strain of *B*. *pseudomallei* (Fig 9). This is likely due to the direct delivery of bacteria to the lung by aerosol. All tissues obtained from AGMs exposed to HBPUB10134a harbored significant tissue burden (Table 3). All AGM exposed to HBPUB10134a had bacteria in their lungs, spleens, livers, kidneys, tracheobronchial lymph nodes (TBLN) and mesenteric lymph nodes (MLN). Three out of five RMs had no bacteria in their lungs, livers or kidneys. Four out of five RMs had bacteria in their spleens. All RMs had bacteria in the TBLN but only three out of five RMs had bacteria in their MLN. The organ bacterial burden in RMs exposed to HBPUB10134a was generally 10-fold lower than in AGMs challenged with an equivalent dose of the same *B*. *pseudomallei* strain, indicating low susceptibility of RMs to infection and less dissemination of bacteria.

AGMs exposed to K96243 was the next group to have high bacterial burdens in most organs/tissues, with bacteria found in lung, spleen, liver, kidney and TBLN in four out of five AGMs (Table 3). The bacterial load in the lungs of AGMs challenged with K96243 was the highest compared to HBPUB10134a and MSHR5855, which is likely due to the higher aerosolized dose of K96243 used in this study (Fig 9). However, one survivor in the K96243-exposed group had no bacteria in blood, urine or any organ.

AGMs exposed to MSHR5855 had the lowest bacterial burdens in organs, with only three out of five lungs harboring bacteria (Table 3). Bacteria were found in two out of five spleens and TBLNs in this group of AGMs. Only one out of five AGMs had bacteria in liver, kidney or MLN, but the bacterial burdens were low and they were in different AGMs. Urine from the MSHR5855-exposed group had no bacteria whereas bacteria were present in two out of three AGMs and four out of five AGMs in the K96243-exposed group and HBPUB10134a-exposed group, respectively. Two of the survivors in the MSHR5855-exposed group did not have bacteria at detectable levels in any organ/tissue or blood. Another survivor in this group had bacteria in lung, TBLN and MLN only. There were statistically significant differences in lung burdens among the AGMs aerosolized with the three different strains of *B*. *pseudomallei* (Fig 9). Bacterial burdens in spleen, liver, kidney and MLN in HBPUB10134a- or K96243-infected AGMs were also statistically different from the organs of the MSHR5855-infected AGMs.

### Histopathology

Since *B*. *pseudomallei* was administered via aerosolization, the lung was the organ with the highest bacterial burden and the most affected organ as shown in bacterial counts from tissue homogenates and histopathology. AGMs exposed to HBPUB10134a had consistently more lesions in multiple organs, a finding that is consistent with disseminated disease compared to RMs exposed to the same strain (S1 Table). This finding provides evidence that AGMs are more susceptible to *B*. *pseudomallei* and accordingly the appropriate species of NHP for the development of an acute model of melioidosis. Development of pathologic lesions in the lungs was seen in AGMs after exposure to all three strains of *B*. *pseudomallei* with the highest severity score noted in AGMs exposed to HBPUB10134a (S2 Table). Development of lesions in multiple organs/tissues was seen in AGMs after inhalation of *B*. *pseudomallei*, HBPUB10134a and K96243, but not in MSHR5855 (S3 Table). The severity score of lesions in both the lungs and disseminated tissues was higher with strain HBPUB10134a compared to strain K96243. Common lung pathology associated with HBPUB10134a-, K96243- and MSHR5855- inhalation were seen in AGM (S1–S3 Figs). In comparison, lung lesions were less severe in RMs after inhalation of *B*. *pseudomalle* HBPUB10134a (S4 Fig and S1 Table). The pathologic lesions in NHPs were consistent with previous studies in animal models and human melioidosis [3,4,11,28–30,35].

### Immunohistochemistry

There was greater and more widely distributed immunoreactivity among the AGM tissues than in RMs exposed to HBPUB10134a. This finding was especially prominent in the lungs (S1C Fig) and the bone marrow (S5 Fig). With the exception of one animal, the lack of immunoreactivity outside of the lungs was striking in the RM group.

Immunoreactivity was present in all of the lung lesions of the AGMs that were exposed to the three strains (S1C, S2C and S3C Figs). Additionally, there was immunoreactivty in all of the disseminated tissues in the AGMs exposed to HBPUB10134a and K96243 (S5–S7 Figs, S2 and S3 Tables). There was no immunoreacitivty in disseminated tissues in AGMs exposed to MSHR5855 except in the one animal with the brain lesion (S8 Fig and S3 Table).

## Discussion

The goal of this study was to identify an acute NHP model of melioidosis that results in significant lethality and also reflects some aspects of acute melioidosis observed in humans. While chronic melioidosis is also a clinically relevant form of the disease, the work described here focused exclusively on developing an acute model. In context of human disease, acute melioiodis is the majority of reported cases (estimated to be greater than 80% of human cases) and results from recent exposure to bacteria [2,43]. Characteristics associated with acute melioidosis include bacteremic pneumonia (depending on the route of infection may be primary or secondary pneumonia) and systemic inflammatory and immune responses observed in multiple organ systems. The formation of large granulomas in various organs is also considered a hallmark of melioidosis disease [2,43].

While we chose to examine AGM and RM, an alternative NHP model using common marmosets (*Callithrix Jacchus*) has been described in the literature [37,39]. Marmosets are exquisitely sensitive to many infections and the same was observed for infection with *B*. *pseudomallei* with an LD_50_ predicted to be less than 10 aerosolized CFUs [37]. Interestingly, even with this notable sensitivity Nelson et al. demonstrated that, strains of *B*. *pseudomallei* exhibited different virulence profiles and pathological features; for example HBPUB10134a was significantly more virulent than K96243 in the marmoset model [39]. Our findings support these observations that identified strain-specific virulence and disease-pathology differences in NHPs. [39]. We examined two additional species of NHP in an attempt to find alternate models for clinical readouts of disease that could be important for the testing and evaluation of medical countermeasures against inhalational melioidosis.

The clinical, immunological and pathological results presented in this report all support the AGM as a model for acute, severe, inhalational melioidosis. RMs are also susceptible to disseminated disease with the most virulent strain HBPUB10134a, but to a notably lesser degree and less consistent extent than the AGM. The RM is likely more suitable for a subacute to chronic model of melioidosis, as this study showed a higher survival rate with only 20% lethality which occurred at a later time point compared to the acute lethal infection seen in AGMs. The comparison of the virulence of the three different strains of *B*. *pseudomallei*, HBPUB10134a, MSHR5855 and high doses of K96243 in AGMs revealed differences in survival rates, time to death, serum levels of cytokines, severity of pathological lesions and bacterial burdens in organs/tissue among these three strains when given at the target doses we used in this work. HBPUB10134a at a comparable dose to MSHR5855 was the most virulent, inducing higher levels of cytokines, chemokines, bacteremia, tissue burdens and acute lethality. AGMs developed acute lethal disease after exposure to aerosolized *B*. *pseudomallei* similar to human acute melioidosis with fever, pneumonia, bacteremia, cytokine response and abscesses in multiple organs. In this study, sera from AGMs exposed to the most virulent strain, HBPUB10134a, revealed higher concentrations of both pro-inflammatory and anti-inflammatory cytokines early and at terminal end points in AGMs when compared to those from RMs. Significantly higher levels of IL-10, IL-1β, IL-1RA, IL-6, IL-8, MCP-1 and MIF were observed in AGMs, but not RMs, at early time points. In addition to these mediators, higher levels of G-CSF, HGF, I-TAC, IFNγ, IP-10, MIG and VEGF were also observed at terminal end points in AGMs but not in RMs. A previous study indicated that leukocytes are the most likely source of these mediators and account for the pathological consequences of melioidosis [42].

Neutrophils and macrophages are phagocytes that play central roles in innate host defense against bacteria due to their ability to detect and eliminate pathogenic microbes [44]. *B*. *pseudomallei* is known to survive and multiply in both human neutrophils and macrophages [45]. These two innate immune cells work in concert to initiate early host defense processes, including phagocytosis, oxidative burst and pro-inflammatory cytokine release upon encounter with *B*. *pseudomallei*. Neutrophils, in particular, are armed with potent bactericidal molecules, including defensins, granule enzymes and proteases [44,46,47]. Early infiltration of neutrophils into the lung is a critical event after inhalation of pathogens and the ability of neutrophils to kill bacteria generally determine the outcome of infection [48]. The pleiotropic cytokine IL-1 is the chief recruiter and activator of neutrophils. IL-1 induces multiple mediators, including the chemokines IL-8 and MCP-1, to direct neutrophils and monocyte/macrophage recruitment, respectively, to sites of infection and injury [49,50]. Additionally, IL-1 and these chemokines activate the antimicrobial activities of the recruited phagocytic cells. Upon pathogen detection with pattern recognition receptors (PRRs), these cells are activated and acquire an inflammatory phenotype to induce IL-1β, TNFα and chemokines to recruit other phagocytic cells for pulmonary defense [51–54].

The processing of IL-1β requires the activation of an inflammasome, an intracellular platform of molecules assembled upon host cell activation, and proteolytic activation of caspase 1 [55,56]. Inflammasomes can be activated by host cell sensing of bacteria or their virulence factors, pathogen-associated molecular patterns (PAMPs), via binding of these PAMPs to toll-like receptors (TLRs) present on the surface of immune cells. Cytosolic PRRs are also capable of detecting intracellular bacteria and PAMPs independent of TLRs [54–57]. Intracellular PRRs such as the nucleotide-binding oligomerization domain (NOD)-like receptors (NLR) activate inflammasomes upon recognition or binding PAMPs including T3SS effectors from *B*. *pseudomallei* [54,55,58–60]. In addition to the proteolytic activation and extracellular release of the IL-1 family of cytokines, inflammasome activation also induces pyroptosis, a lytic form of cell death, through the action of activated caspase 1 [61].

IL-1β induces other cytokines including IL-1β itself, IL-1RA, TNFα, IL-6, and chemokines and adhesion molecules to promote early recruitment of neutrophils and other immune cells to infection sites [49,62,63]. The pro-inflammatory action of IL-1 includes the induction of acute phase proteins and procoagulant factor (tissue factor), which drives vascular inflammation. The IL-1 family of cytokines includes IL-1RA, a natural antagonist of IL-1 that binds IL-1 receptor 1 (IL-1R1) without signal transduction and blocks IL-1 from binding to IL-1R1 [49,62,63]. All three strains of *B*. *pseudomallei* induced high levels of IL-1RA early in the disease course, and levels remained high at terminal end points except for the terminal end points of MSHR5855-infected AGMs. Levels of IL-1RA followed similar patterns as IL-1β in this study across all strains of *B*. *pseudomallei*. The induction of IL-1RA is a self-regulatory host response to downregulate the deleterious inflammatory effects of IL-1β. However, IL-1RA has a short half-life *in vivo* and the high levels seen in this study may not be sufficient to block the effects of IL-1β. TNFα, the prototypical inflammatory cytokine required to control bacterial infection, was not detected in all NHPs in this study.

IFNγ is another critical cytokine for host defense against *B*. *pseudomallei* in mouse models of infection and *in vitro* [24,26,28,30]. IFNγ activates monocytes/macrophages to enhance their microbicidal activities and has synergistic effects with both IL-1 and TNFα [49]. The host protective effect of IFNγ is well-known; IFNγ-deficient C57BL/6 mice were acutely susceptible to pulmonary *B*. *pseudomallei* infection compared to normal C57BL/6 mice [64]. In this study, we showed a higher serum level of IFNγ in the highly susceptible AGM compared to the less susceptible RM after *B*. *pseudomallei* HBPUB10134a infection. The higher levels of IFNγ at terminal time points also correlated with non-survivors of *B*. *pseudomallei* infection in the NHP model presented here. The early activation of macrophages to an “inflammatory” phenotype by cytokines such as IL-1 and IFNγ is critical for host defense [48,51]. IFNγ induces the CXC chemokines, MIG, IP-10 and I-TAC, from multiple cell types to attract T cells and to promote host resistance to bacterial infections [49,65]. MIG, IP-10 and I-TAC were observed with all three strains of *B*. *pseudomallei* at both time points. Relatively higher levels of IFNγ were induced by HBPUB10134a than the other two strains of *B*. *pseudomallei*, perhaps indicating pathogenic differences between bacterial strains.

MCP-1 and IL-8 are chemokines which can be induced by LPS, mitogens, bacterial superantigens, IL-1, TNFα and pathogens [49,65]. MCP-1 and IL-8 recruit blood monocytes/macrophages and neutrophils, respectively, to the lung after inhalation of pathogens and these recruited cells become activated and are highly inflammatory. MCP-1 was observed at early time points with all three strains of *B*. *pseudomallei* and increased to higher levels at terminal end points for HBPUB10134a and K96243. IL-8 was induced by HBPUB10134a and K96243 at early time points and reached a higher level at terminal end points. Interestingly, IL-8 was not induced by MSHR5855 in this study which seemingly contradicts the host protective effect of neutrophils.

IL-6 has multiple biological activities on a variety of cells and stimulates both B cells and T cells [66]. IL-1β is a potent inducer of IL-6 and the extremely high levels of IL-6 induced by *B*. *pseudomallei* shown in this study likely reflect the multiple cell sources of IL-6. IL-6 also regulates immune responses by antagonizing TGFβ and interferes with the repair function of macrophage [67]. In this study, elevated CRP which is induced by IL-6, was present after infection with all three strains of *B*. *pseudomallei*. HGF (hepatocyte growth factor) is induced by liver cells upon liver injury and trauma and is partially responsible for the high regenerative capacity of the liver [68]. We showed for the first time in this study, increased levels of HGF with all three strains of *B*. *pseudomallei* at both time points. Interestingly, elevated IL-6 and HGF levels were significantly correlated with non-survivors of *B*. *pseudomallei* infection.

G-CSF regulates hematopoietic stem cell activity including granulopoiesis [69]. Together with IL-1 and IL-6, G-CSF promotes neutrophil growth and survival. In response to infection and stress, G-CSF is induced in the macrophage to stimulate neutrophil production and release from the bone marrow [69]. Elevated G-CSF levels were also found in non-survivors of *B*. *pseudomallei* infection in this study.

IL-10 is an anti-inflammatory cytokine that plays a major role in downregulating immune responses [70]. IL-10 deactivates macrophages/monocytes by downregulating MHC class II and costimulatory receptor expression on these cells, resulting in lower microbicidal activities in macrophages. In the present study, we report for the first time, elevated IL-10 in sera at both time points in AGMs infected with *B*. *pseudomallei* HBPUB10134a. Increases of IL-10 were observed only at terminal end points with *B*. *pseudomallei* K96243 and MSHR5855. This suggests that early IL-10 induction is associated with virulence of *B*. *pseudomallei* as a mechanism to evade host defense.

We identified several elevated mediators (G-CSF, HGF, IFNγ, IL-1β, IL-1RA, IL-6, I-TAC, MCP-1, MIG, MIP-1β) at terminal end points of inhalational *B*. *pseudomallei* infection in AGMs, which were significantly correlated to mortality. IL-1β, IL-6, IL-8, MCP-1 and IFNγ, were previously reported to be elevated in lethal infection using a high dose of *B*. *pseudomallei* 1026b in AGMs [35]. However, higher levels of IL-1β and IL-6 were also observed in RMs exposed to 1026b in that study. Elevated IL-6 and IL-8 were also detected in sera of human acute cases of melioidosis, with elevated IL-6 identified as the best predictor of mortality [71,72]. Another study identified elevated IFNγ and its inducers, IL-18, IL-12p40 and IL-15 in blood culture-positive melioidosis patients [73]. Overall, the response of RMs to HBPUB10134a was more variable, with only 20% mortality, although survivors also harbored bacteria in multiple organs even at day 47 PI. The lower bacterial burdens, fewer cases of bacteremia, and reduced pathological lesions in RMs suggest chronic or subacute infection, possibly resembling human chronic melioidosis. There are subtle differences in comparative virulence of *B*. *pseudomallei* strains in AGMs in this study compared with previous mouse models using these *B*. *pseudomallei* strains from two geographical locations. MSHR5855 was comparatively less virulent than HBPUB10134a at approximately the same dose in AGMs, with 60% survival at the end of the study period. Respiratory deterioration was noted in these AGMs similar to those infected with HBPUB10134a and K96243. When delivered at a higher target dose, the demonstrably less virulent strain K96243, was similarly virulent compared to HBPUB10134a, and induced high levels of immune mediators comparable to those of HBPUB10134a, and had a 20% survival rate.

Another important factor that regulates phagocyte phenotypic function is the types of dead cells it encounters. The modulation of different forms of host cell death by various bacteria and perhaps even different strains of *B*. *pseudomallei* might explain the differences in phagocyte recruitment and mechanism of pathogenicity [74–76]. Recruited and activated neutrophils produce ROS and proteases, including metalloproteases (MMPs) and granule proteinase 3 (PR3). While this is protective in the early phase of bacterial infection, an overabundance of neutrophils can promote organ damage during the resolving phase or contraction phase of bacterial infection. Neutrophils have a short lifespan of 5 to 48 hours and die by apoptosis under normal homeostatic conditions. However, at sites of infection, the neutrophil lifespan increases due to the high levels of inflammatory cytokines, such as IL-1, TNFα, IL-6 and G-CSF, present in the local tissue environment [77]. Macrophages are central to the efficient clearance of apoptotic cells including neutrophils and cellular debris to promote resolution of disease at the end of an infection [78,79]. The uptake of apoptotic cells leads to anti-inflammatory effects and dampening of anti-microbial function in the efferocytosing cell to promote tissue repair and return to homeostasis [79,80]. Although the induction of IL-1β is beneficial to host defense, the simultaneous inflammasome activation by PRRs is detrimental as it induces macrophage pyroptosis, releasing cellular contents and DAMPs. Pyroptosis of macrophages was reported in murine melioidosis [60,61]. The prolonged lifespan of neutrophils may allow *B*. *pseudomallei* to replicate inside neutrophils and use them as “Trojan horses” to transport bacteria to an efferocytosing cell and promote bacterial spread [76]. Moreover, unremoved apoptotic neutrophils usually proceed to secondary necrosis, releasing potent granule proteases, lytic enzymes and other DAMPs to promote tissue destruction [75]. The neutrophil protease PR3 can also proteolytically process and release IL-1β, creating a vicious cycle of neutrophil recruitment and inflammation. Thus, the inflammatory cytokines IL-1 and IL-6 induced by *B*. *pseudomallei* modulate different forms of cell death in addition to activating phagocytes. Necrotic cell death also expels and releases bacteria at infection sites. Necrotic lesions were seen in histopathology and the severity of lesions in various organs also correlates with the virulence of the *B*. *pseudomallei* strains in this study. A previous study reported granule protease expression in human melioidosis patients, which suggests lytic neutrophil cell death with human *B*. *pseudomallei* infection [81].

Most studies indicate that *B*. *pseudomallei* is resistant to intracellular killing by both neutrophils and macrophages unless activated by IFNγ [64,82]. However, both cell types are required to control *B*. *pseudomallei* infection, as shown by the increase in lethality with the depletion of either cell type in mouse infection models [83,84]. *B*. *pseudomallei* evades ROS killing in phagosomes and escapes the phagolysosomes of phagocytes via the type III secretion cluster 3 (T3SS3) effector BopA and the translocator BipD [85]. Despite substantial advances made in the evasion mechanisms used by *B*. *pseudomallei* and discoveries of novel bacterial virulence factors, there are essential gaps in knowledge of why phagocytes are unable to efficiently kill *B*. *pseudomallei* and allow bacteria to persist in the more favorable cytosolic environment of both neutrophils and macrophages. Since *B*. *pseudomallei* can replicate intracellularly in both neutrophils and macrophages, the bidirectional interaction of these cells may be the weak link of host response targeted by *B*. *pseudomallei* [76]. The inability to clear intracellular *B*. *pseudomallei* promotes intracellular persistence and high organ burdens. The prolonged lifespan of neutrophils allow *B*. *pseudomallei* to replicate and they then use neutrophils as transport vehicles to migrate to multiple organs. Uptake of these infected neutrophils by the efferocytosing macrophage facilitates transfer of *B*. *pseudomallei* to another cell and thus promotes the spread of bacteria to another cell. The early release of IL-1β is important for recruitment of neutrophils, but prolonged IL-1β release also has pathological consequences as IL-1β recruits more neutrophils to infection sites and inflammasome activation leads to immflammatory pyroptotic macrophage cell death. Neutrophils are more resistant to pyroptosis than macrophages but unremoved, infected neutrophils die by “secondary necrosis”. Necrotic neutrophils cause tissue damage by releasing DAMPs, granule proteases, MMPs, PAMPs and bacteria.

*B*. *pseudomallei* HBPUB10134a and MSHR5855 are highly virulent strains based on mouse models of infection by both aerosol and intraperitoneal routes [28–30]. Both were isolated from tracheal sputum samples but were from different geographic regions. HBPUB10134a originated from Thailand, whereas MSHR5855 came from a patient in Australia. K96243, a relatively weaker strain in mouse models of infection, was an isolate from a diabetic Thai patient with septic infection who died from acute melioidosis [40]. In this study, AGMs were challenged with a higher dose of K96243 in order to increase the likelihood of observing clinical signs induced by this less virulent strain of *B*. *pseudomallei*. This dose differential likely resulted in the lower survival rate of K96243 compared to MSHR5855. These data demonstrate that decreased virulence associated with less virulent strains can be overcome by increasing the delivered dose.

It is imperative to reiterate that these inter-bacterial strain differences noted in this work are based on delivering different lethal dose equivalents to induce clinical signs of disease. While admittedly the major strain difference revolves around bacterial lethality, we can discern pathological and clinical signs that are strain-specific in context of differential inhaled doses. However, HBPUB10134a is clearly more virulent than MSHR5855 in AGMs even when delivered at very comparable delivered doses of aerosolized bacteria. Strain specific virulence factors might account for the pathogenicity of *B*. *pseudomallei* in different animal models. LPS, the best-known virulence factor of gram-negative bacteria, binds TLR4 on macrophages and activates NFκB to induce pro-inflammatory cytokines. Most strains from Thailand and Australia have LPS type A, a weaker inducer of inflammatory cytokines than LPS type B [86]. All three isolates of *B*. *pseudomallei* have type A LPS [87]. *Burkholderia* intracellular motility factor A (BimA) and filamentous hemagglutinin gene (*fhaB3*) were previously identified as significant variable virulence factors based on their geographic locations [2,88]. BimA_Bp_ was associated with isolates from Thailand, whereas 12% of isolates from Australia have the BimA_Bm_ variant, which shares only 54% homology with BimA_Bp_. Using clinical data from Australian melioidosis cases, the BimA_Bm_ gene was reported to have a strong association with neurological disease [88]. Two NHPs infected with MSHR5855 were observed to have neurological changes to include behavioral alterations, limited mobility on one-side, and generalized weakness. Upon necropsy, the NHP observed to have decreased usage of its appendages had noted neuroanatomical changes including a lesion and multifocal hemorrhaging in the right cerebrum and encephalitis with positive IHC staining for *B*. *pseudomallei*. All three *B*. *pseudomallei* strains used in this study have BimA_Bp_ [88]. Another variable virulence factor present in *B*. *pseudomallei* strains from different geographical locations is the filamentous hemagglutinin (FhaB) [88]. *B*. *pseudomallei* strains lacking *fhaB3* are significantly associated with non-severe cutaneous disease when compared to strains with *fhaB3* [88]. Another genomic difference among strains from these two geographical locations is the flagellum biosynthesis cluster. The *B*. *thailandensis*-like flagellum and chemotaxis (BTFC) gene cluster is dominant in Australian isolates whereas the *Yersinia*-like fimbrial (YLF) gene cluster is associated with Thai strains. The BTFC and YLF gene clusters are mutually exclusive in the genome of *B*. *pseudomallei* and isolates with YLF are associated with clinical cases of melioidosis. Strain K96243 from Thailand was shown to possess the YLF cluster [89]. While further work is required to understand the potential strain-specific neuro-tropisms, these data are intriguing and are suggestive of recapitulation of human disease.

In conclusion, AGMs are highly susceptible to *B*. *pseudomallei* HBPUB10134a and high doses of K96243. MSHR5855 is appreciably less virulent than HBPUB10134a at approximately the same dose in AGMs and the MSHR5855-infected group displayed neuro-involvement not seen in AGMs infected with the other two strains from Thailand. Infection with MSHR5855 in AGM appears to mimic the neurological melioidosis cases seen in Australia. AGMs infected with HBPUB10134a and K96243 (delivered at approximately three times the dose of HBPUB10134a) had similar mean times to death, with 0% and 20% survival, respectively. High levels of cytokines and chemokines were also evident with *B*. *pseudomallei-*infected AGMs, which is similar to acute melioidosis in humans. AGMs failed to control *B*. *pseudomallei* infection, as indicated by the high bacterial burden in various organs. Multi-organ injury occurred, leading to high mortality in AGMs. In this study, pro-inflammatory and anti-inflammatory cytokines, as well as cell- and organ-regenerative mediators are correlated with lethal melioidosis. A normal host response to infection relies on macrophage activation to kill pathogens and to repair tissue damage caused by the elimination of microbes. The return to homeostasis is critical for host survival and the balance of these macrophage phenotypes, kill verus repair, is dependent on the uptake of apoptotic cells, proinflammatory cytokines and the type of dead cells encountered. Phagocytosis and clearance of apoptotic cells (efferocytosis) leads to suppression of immune responses through TGFβ activation [80]. However, high levels of IL-6 at the resolution stage downregulate TGFβ and prevent resolution of infection/disease [67]. Necrotic cells and pyropytotic cells release DAMPs to further activate PRRs resulting in a vicious cycle of inflammatory stimuli and activation of proinflammatory cytokines, IL-1β and IL-6. The sustained high levels of these two cytokines at the terminal end point of AGM exposed to all three strains of *B*. *pseudomallei* shown in our study prevents macrophages from switching to a “healing” response. This dysregulated immune response of an overabundance of IL-1β and IL-6 at terminal end points and inflammatory cell death contributes to the clinical picture of melioidosis in AGMs.

### Disclaimer

Opinions, interpretations, conclusions, and recommendations are those of the author and are not necessarily endorsed by the U.S. Army.

## Supporting information

S1 Fig Gross and histologic lung lesions of AGM exposed to *B*. *pseudomallei* HBPUB10134a. (A) Lungs: there are multiple, sometimes coalescing, flat white to tan nodules surrounded by a hyperemic rim of congestion. (B) Lung: Pneumonia, necrosuppurative and fibrinous with edema and hemorrhage, 4x, H&E. * denotes pyogranuloma. (C) Lung: There is widespread immunoreactivity (brown staining) in the area of inflammation and necrosis, 4X, IHC antibody *B*. *psuedomallei*.
(TIF)

S2 Fig Gross and histologic lung lesions of AGM exposed to *B*. *pseudomallei* K96243. (A) Lungs: Multiple white to tan nodules that are surrounded by a hyperemic rim of congestion. “a” marks alveolar lumina. (B) Lung: Bronchopneumonia, necrosuppurative, multifocal, moderate with hemorrhage, fibrin and edema, 4x, H&E. (C) Immunoreactive (brown staining) inflammatory cells and necrotic debris within alveolar lumina, 4x, IHC antibody *B*. *pseudomallei*. “a” marks alveolar lumina.
(TIF)

S3 Fig Gross and histologic lung lesions of AGM exposed to *B*. *pseudomallei* MSHR5855. (A) Lung: Multiple pyogranulomas with pleural fibrosis, 2x, H&E. “a” marks alveolar lumina. (B) Immunoreactive (brown staining) inflammatory cells and necrotic debris within center of pyogranuloma, 10x, IHC antibody *B*. *pseudomallei*. “*” denotes pyogranuloma; “a” marks alveolar lumina. (C) Immunoreactive (brown staining) inflammatory cells and necrotic debris, 40x, IHC antibody *B*. *pseudomallei*.
(TIF)

S4 Fig Gross and histologic lung lesions of RM exposed to *B*. *pseudomallei* HBPUB10134a. (A) Lung: There are multiple solitary to coalescing tan nodules. “*” denotes abscess. (B) Lung: There are multifocal immunoreactive (brown staining) inflammatory cells and necrotic cellular debris within an abscess, 20x, IHC antibody *B*. *psuedomallei*.
(TIF)

S5 Fig Histologic bone marrow lesions of AGM exposed to *B*. *pseudomallei* HBPUB10134a. (A) Bone marrow: Multifocal pyogranulomas, 10x, H&E. (B) Bone marrow: there is widespread immunoreactivity (brown staining) within the pyogranuloma, 40x, IHC antibody *B*. *psuedomallei*. “*” denotes pyogranuloma.
(TIF)

S6 Fig Histologic liver lesions of AGM exposed to *B*. *pseudomallei* K96243. (A) Liver: Multiple white nodules. (B) Liver: Multiple pyogranulomas, 2x, H&E. “*” denotes pyogranuloma; “b” marks portal area.
(TIF)

S7 Fig Histologic kidney lesions of AGM exposed to *B*. *pseudomallei* K96243. (A) Kidney: Multifocal moderate neutrophilic and histiocytic nephritis, 4x, H&E. “c” marks glomeruli. (B) Kidney: Immunoreactive (brown staining) inflammatory cells and necrotic debris, 4x, IHC antibody *B*. *pseudomallei*.
(TIF)

S8 Fig Histologic brain lesions of AGM exposed to *B*. *pseudomallei* MSHR5855. (A) Brain, cerebrum, right hemisphere: Multifocal hemorrhage and cavitation with a locally extensive increase in size. (B) Cerebrum, right hemisphere: Multifocal moderate neutrophilic and histiocytic encephalitis is marked with “d”, 2x, H&E. (C) Cerebrum, right hemisphere: Immunoreactive (brown staining) inflammatory cells and necrotic debris, 2x, IHC antibody *B*. *pseudomallei*.
(TIF)

S1 Table Histopathologic findings in AGMs and RMs following inhalation of *B*. *pseudomallei* HBPUB10134a. (PDF)

S2 Table Histopathological and IHC score in AGM’s target organ post-exposure to *B*. *pseudomallei*. (PDF)

S3 Table Histopathological and IHC score in disseminated organs of AGM post-exposure to *B*. *pseudomallei*. (PDF)
