# Supplementary figures and images for: Comparative virulence of three different strains of Burkholderia pseudomallei in an aerosol non-human primate model
Source: PLoS Negl Trop Dis. 2021 Feb 11;15(2):e0009125. doi: 10.1371/journal.pntd.0009125 (PMC7904162; doi:10.1371/journal.pntd.0009125)

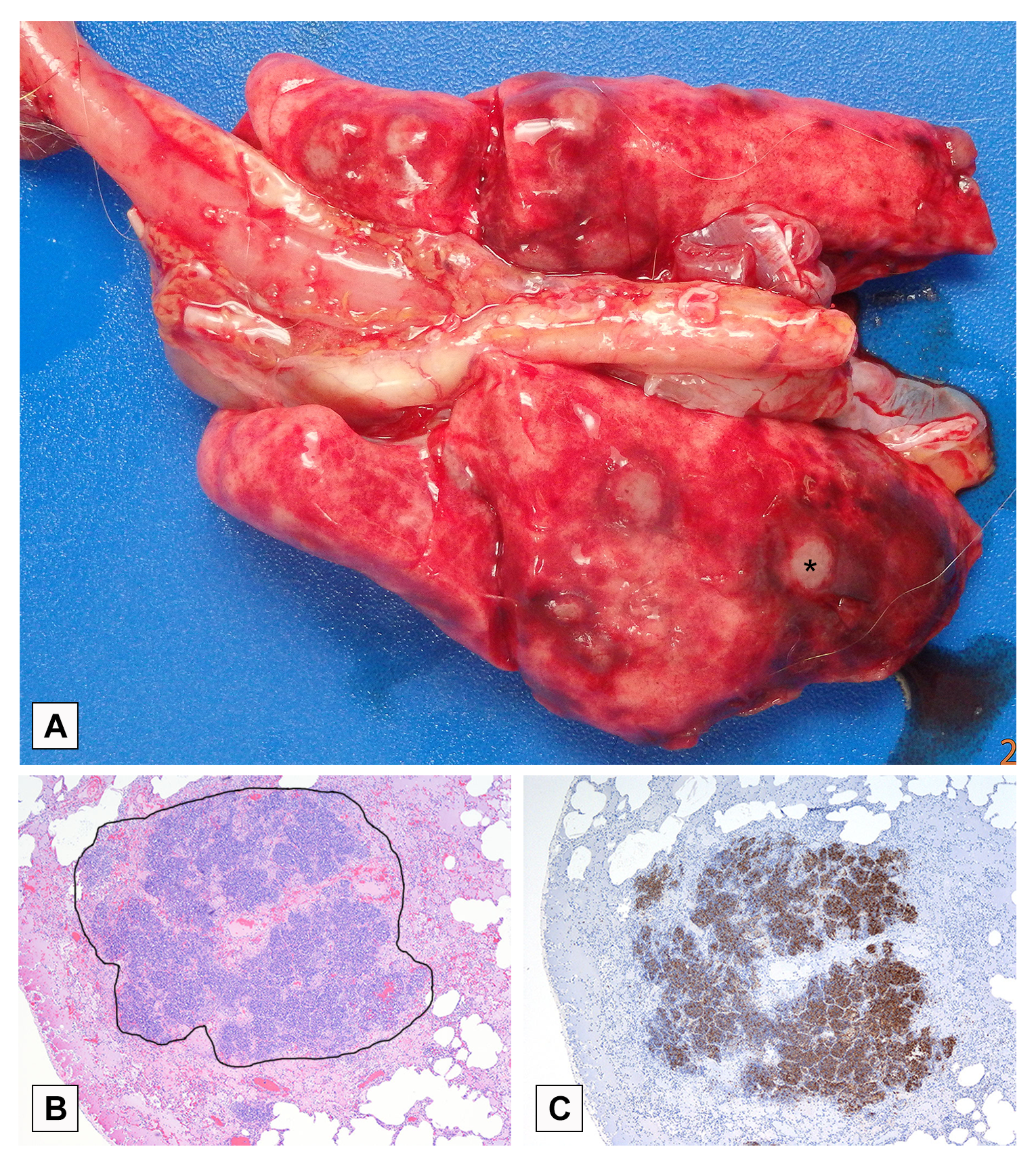

Supplement: S1 Fig — (A) Lungs: there are multiple, sometimes coalescing, flat white to tan nodules surrounded by a hyperemic rim of congestion. (B) Lung: Pneumonia, necrosuppurative and fibrinous with edema and hemorrhage, 4x, H&E. * denotes pyogranuloma. (C) Lung: There is widespread immunoreactivity (brown staining) in the area of inflammation and necrosis, 4X, IHC antibody B. psuedomallei. (TIF) [file pntd.0009125.s001.tif]

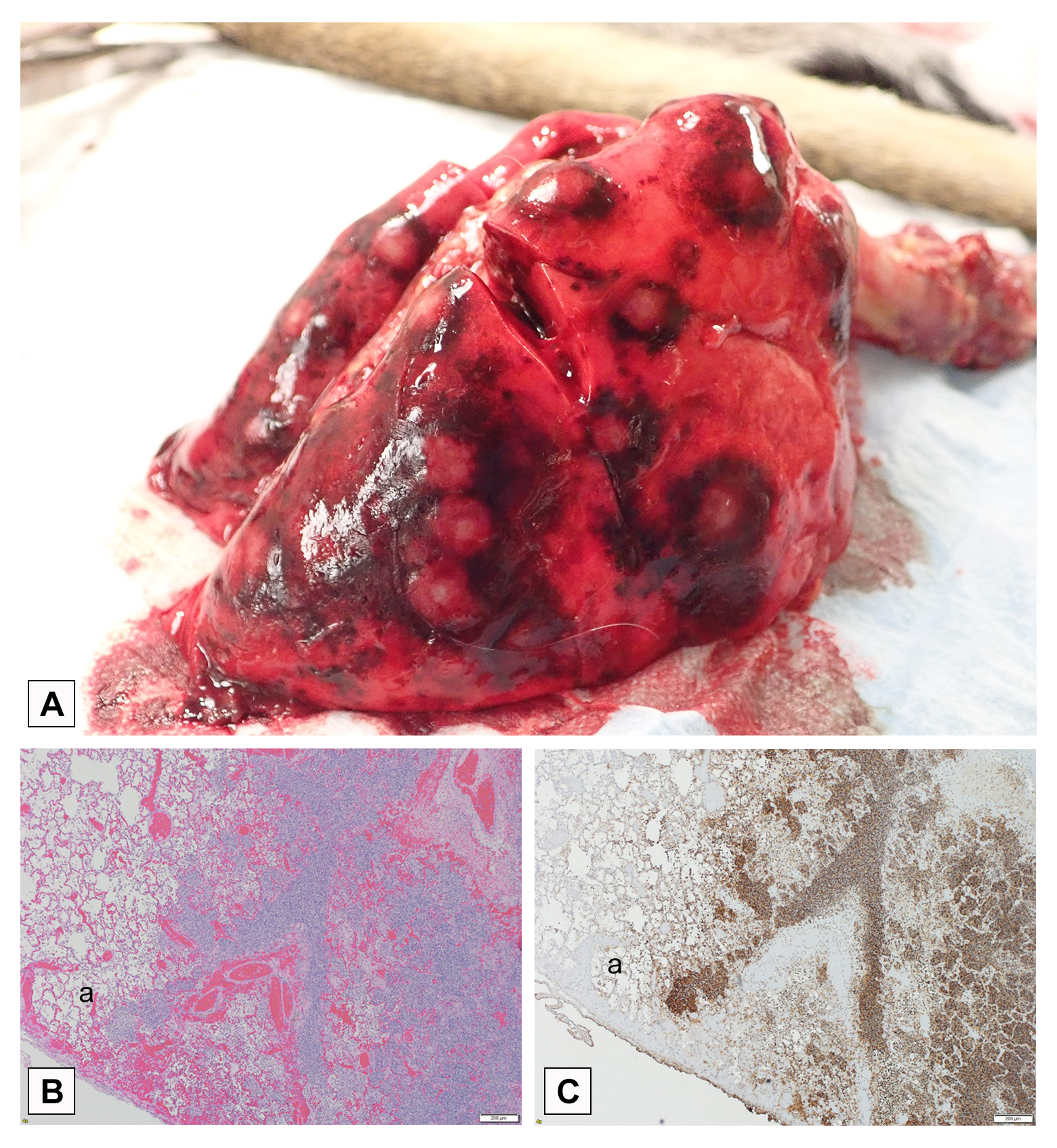

Supplement: S2 Fig — (A) Lungs: Multiple white to tan nodules that are surrounded by a hyperemic rim of congestion. “a” marks alveolar lumina. (B) Lung: Bronchopneumonia, necrosuppurative, multifocal, moderate with hemorrhage, fibrin and edema, 4x, H&E. (C) Immunoreactive (brown staining) inflammatory cells and necrotic debris within alveolar lumina, 4x, IHC antibody B. pseudomallei. “a” marks alveolar lumina. (TIF) [file pntd.0009125.s002.tif]

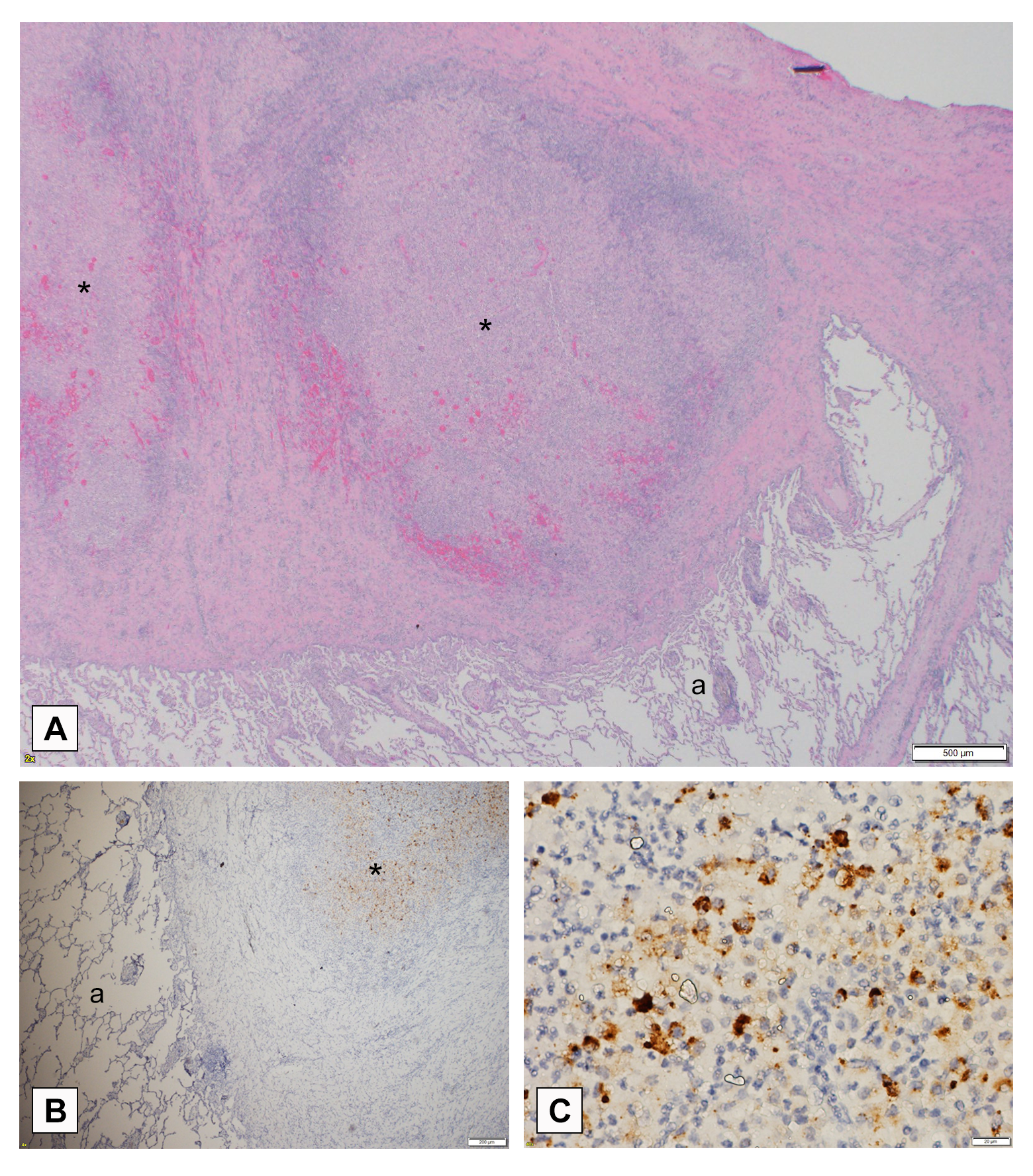

Supplement: S3 Fig — (A) Lung: Multiple pyogranulomas with pleural fibrosis, 2x, H&E. “a” marks alveolar lumina. (B) Immunoreactive (brown staining) inflammatory cells and necrotic debris within center of pyogranuloma, 10x, IHC antibody B. pseudomallei. “*” denotes pyogranuloma; “a” marks alveolar lumina. (C) Immunoreactive (brown staining) inflammatory cells and necrotic debris, 40x, IHC antibody B. pseudomallei. (TIF) [file pntd.0009125.s003.tif]

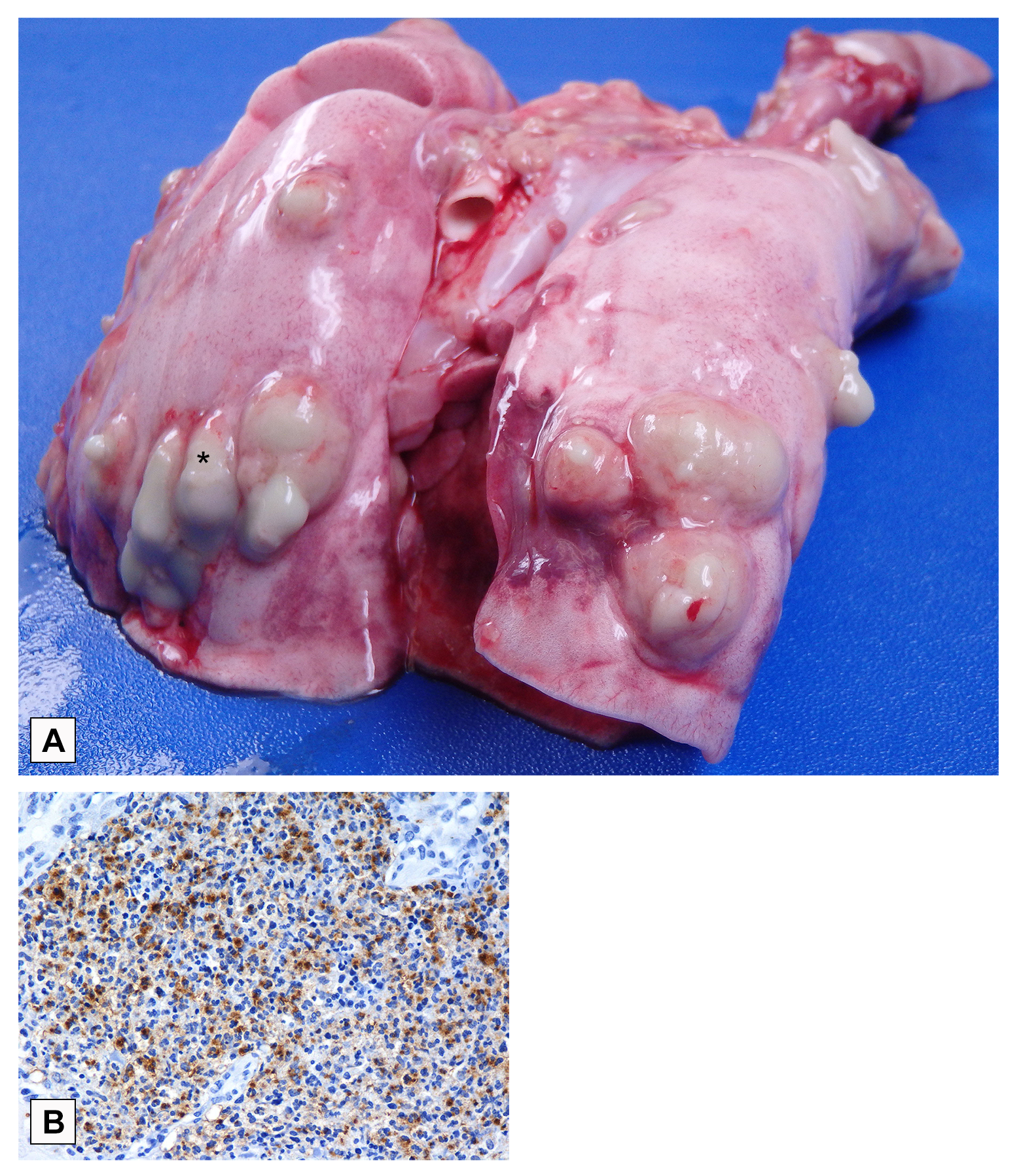

Supplement: S4 Fig — (A) Lung: There are multiple solitary to coalescing tan nodules. “*” denotes abscess. (B) Lung: There are multifocal immunoreactive (brown staining) inflammatory cells and necrotic cellular debris within an abscess, 20x, IHC antibody B. psuedomallei. (TIF) [file pntd.0009125.s004.tif]

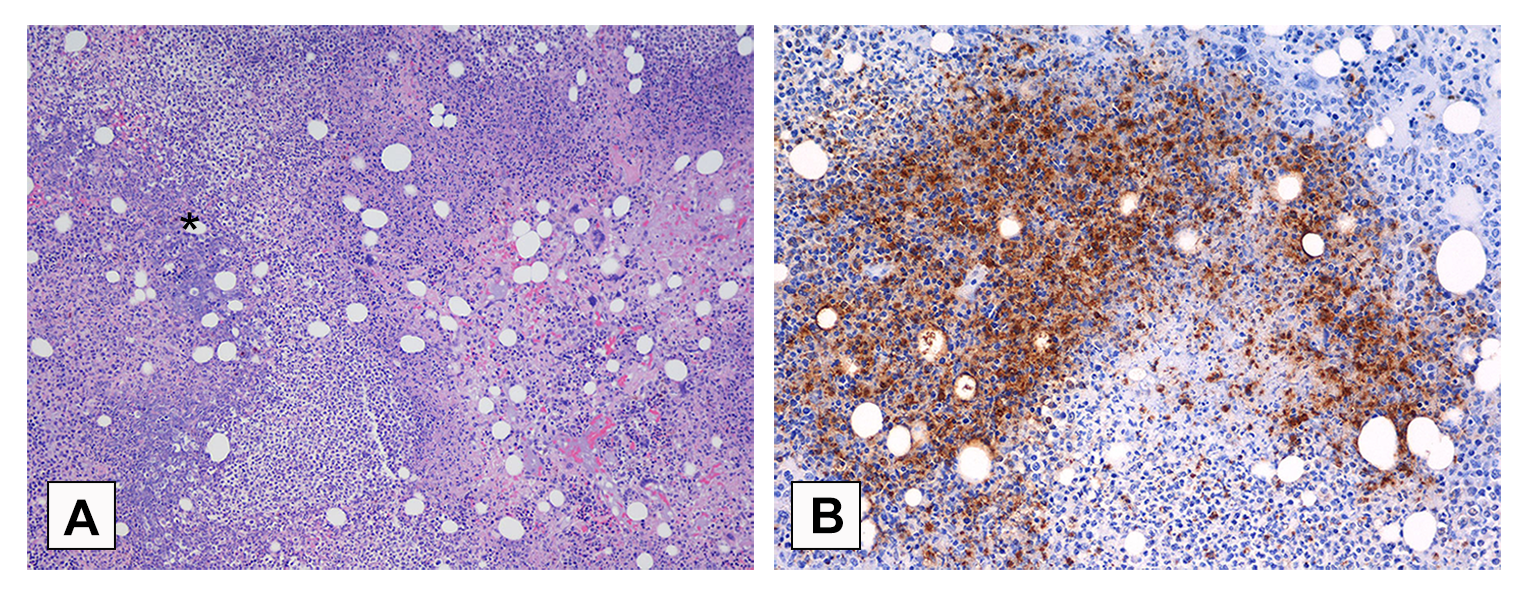

Supplement: S5 Fig — (A) Bone marrow: Multifocal pyogranulomas, 10x, H&E. (B) Bone marrow: there is widespread immunoreactivity (brown staining) within the pyogranuloma, 40x, IHC antibody B. psuedomallei. “*” denotes pyogranuloma. (TIF) [file pntd.0009125.s005.tif]

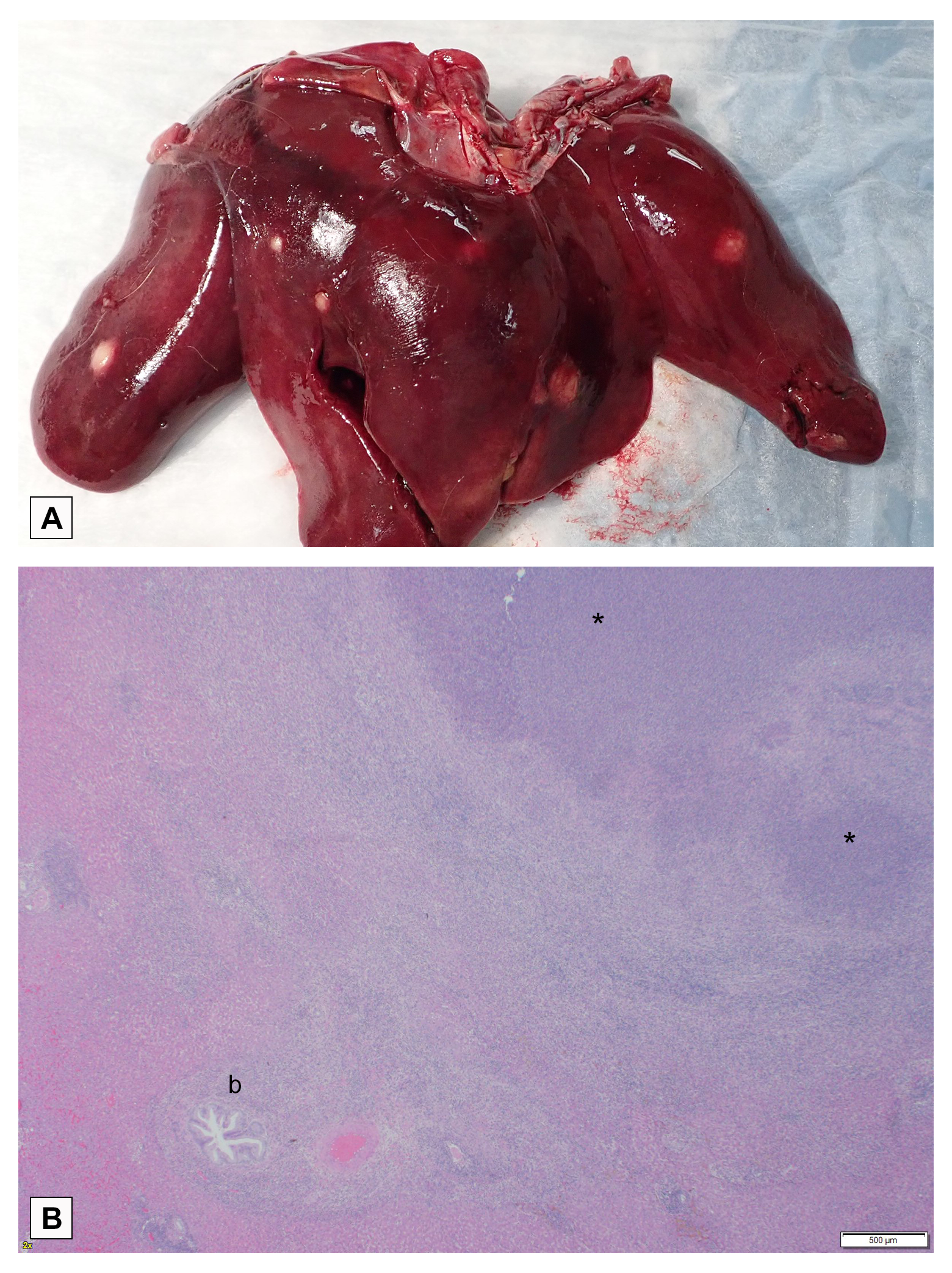

Supplement: S6 Fig — (A) Liver: Multiple white nodules. (B) Liver: Multiple pyogranulomas, 2x, H&E. “*” denotes pyogranuloma; “b” marks portal area. (TIF) [file pntd.0009125.s006.tif]

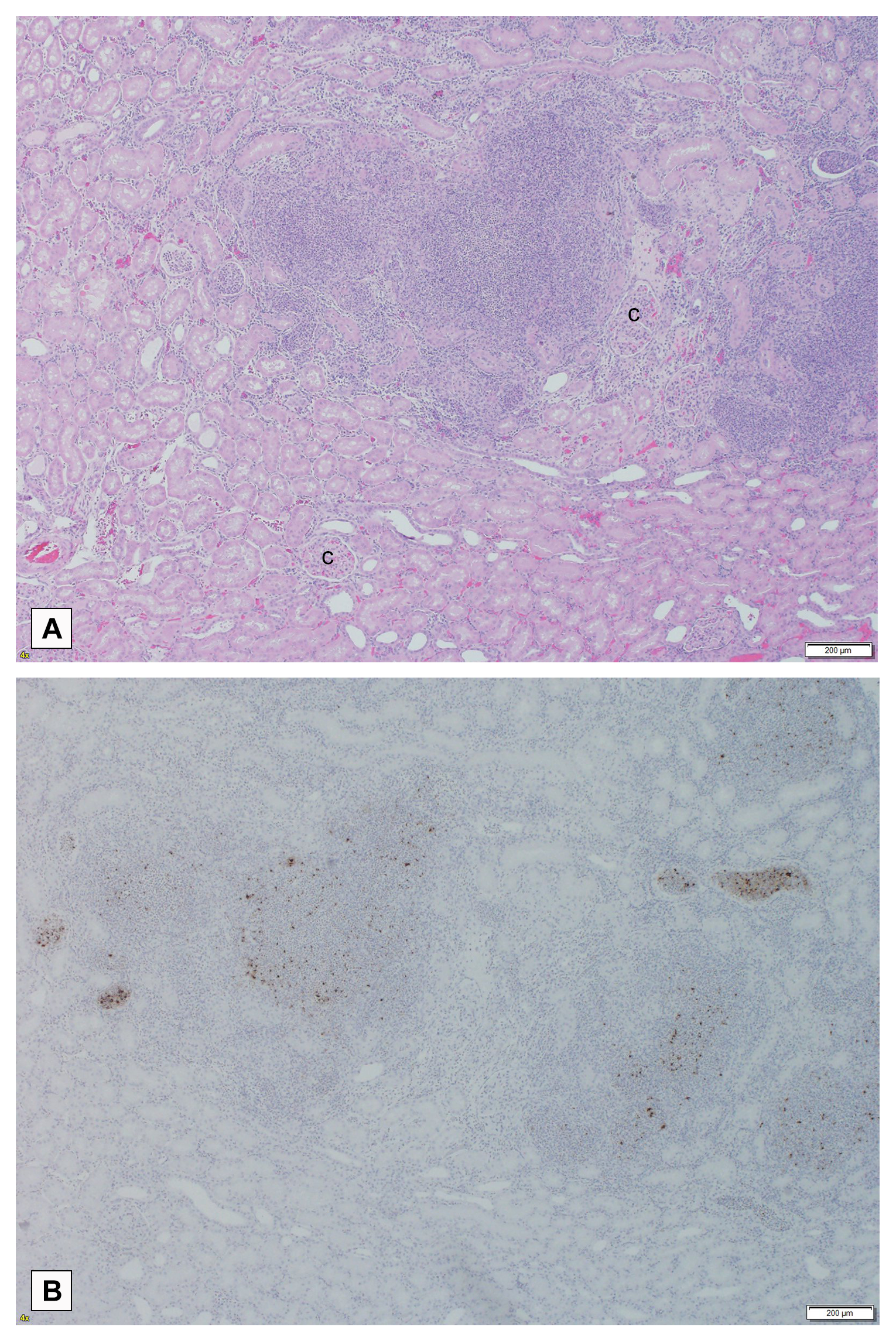

Supplement: S7 Fig — (A) Kidney: Multifocal moderate neutrophilic and histiocytic nephritis, 4x, H&E. “c” marks glomeruli. (B) Kidney: Immunoreactive (brown staining) inflammatory cells and necrotic debris, 4x, IHC antibody B. pseudomallei. (TIF) [file pntd.0009125.s007.tif]

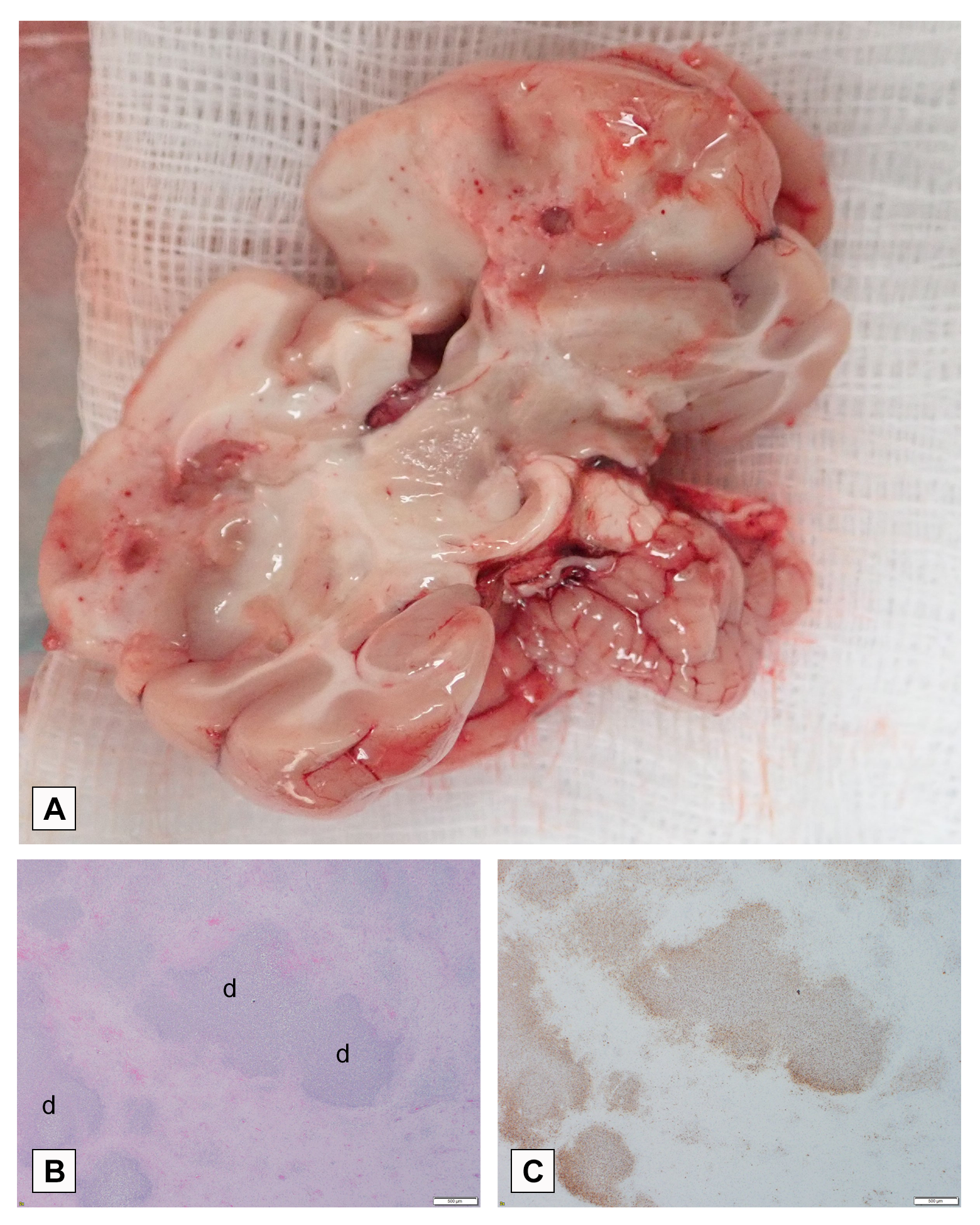

Supplement: S8 Fig — (A) Brain, cerebrum, right hemisphere: Multifocal hemorrhage and cavitation with a locally extensive increase in size. (B) Cerebrum, right hemisphere: Multifocal moderate neutrophilic and histiocytic encephalitis is marked with “d”, 2x, H&E. (C) Cerebrum, right hemisphere: Immunoreactive (brown staining) inflammatory cells and necrotic debris, 2x, IHC antibody B. pseudomallei. (TIF) [file pntd.0009125.s008.tif]
