# Supplementary material for: Comparative virulence of three different strains of Burkholderia pseudomallei in an aerosol non-human primate model
Source: PLoS Negl Trop Dis. 2021 Feb 11;15(2):e0009125. doi: 10.1371/journal.pntd.0009125 (PMC7904162; doi:10.1371/journal.pntd.0009125)
Supplement: S1 Table — (PDF) [file pntd.0009125.s009.pdf]

S1 Table. Histopathologic findings in AGMs and RMs following inhalation of *B. pseudomallei* HBPUB10134a.

	Prevalance		Severity[a]: average (range)	
	**AGM**	**RM**	**AGM**	**RM**
**Pneumonia**	5/5	3/5	3.8 (3-5)	3.3 (2-4)
**Pleuritis**	4/5	2/5	3.7 (3-5)	3 (2-4)
**Pulmonary pyogranulomas**	0/5	1/5	N/A	4 (4)
**Hepatitis**	5/5	3/5	2.4 (2-3)	2 (2-3)
**Splenitis**	5/5	4/5	4.4 (3-4)	2.5 (2-3)
**Lymphadenitis**				
**Tracheobronchial**	3/4[b]	2/5	2.6 (2-3)	2 (2)
**Mediastinal**	4/5	1/5	3.2 (3-4)	2 (2)
**Nephritis**	0/5	2/5	N/A	1.5 (1-2)
**Myelitis (bone marrow)**	1/5	0/5	1 (1)	N/A
**Bone marrow pyogranulomas**	4/5	0/5	2.5 (2-4)	N/A

[a]Severity scores: 1: minimal, 2: mild, 3: moderate, 4: marked, 5: severe.

[b]One tracheobronchial lymph node was not examined.
