# Supplementary material for: Comparative virulence of three different strains of Burkholderia pseudomallei in an aerosol non-human primate model
Source: PLoS Negl Trop Dis. 2021 Feb 11;15(2):e0009125. doi: 10.1371/journal.pntd.0009125 (PMC7904162; doi:10.1371/journal.pntd.0009125)
Supplement: S2 Table — (PDF) [file pntd.0009125.s010.pdf]

S2 Table. Histopathological and IHC score in AGM's target organ post-exposure to *B. pseudomallei*.

Lesion	HBPUB10134a			K96243			MSHR5855		
	Prevalance	Severity[a] Average (range)	IHC	Prevalance	Severity[a] Average (range)	IHC	Prevalance	Severity[a] Average (range)	IHC
**Lung**									
Pneumonia	5/5	3.8 (3-5)	+	4/5	3 (2-4)	+	0/5	N/A	N/A
Pleuritis	4/5	3.7 (3-5)	+	0/5	N/A	N/A	0/5	N/A	N/A
Pyogranulomas	0/5	N/A	N/A	1/5	2	+	1/5	3	+
Pleural fibrosis	0/5	N/A	N/A	1/5	2	+	1/5	3	+

[a]Severity scores: 1: minimal, 2: mild, 3: moderate, 4: marked, 5: severe
