# Supplementary material for: Comparative virulence of three different strains of Burkholderia pseudomallei in an aerosol non-human primate model
Source: PLoS Negl Trop Dis. 2021 Feb 11;15(2):e0009125. doi: 10.1371/journal.pntd.0009125 (PMC7904162; doi:10.1371/journal.pntd.0009125)
Supplement: S3 Table — (PDF) [file pntd.0009125.s011.pdf]

S3 Table. Histopathological and IHC score in disseminated organs of AGM post-exposure to *B. pseudomallei*.

	HBPUB10134a			K96243			MSHR5855		
**Lesion**	**Prevalance**	**Severity[a] Average (range)**	**IHC**	**Prevalance**	**Severity[a] Average (range)**	**IHC**	**Prevalance**	**Severity[a] Average (range)**	**IHC**
**Splenitis**	5/5	4.4 (3-4)	+ (5/5)	2/5	1.5 (1-2)	+ (2/2)	0/5	N/A	N/A
**Hepatitis**	5/5	2.4 (2-3)	+ (3/5)	2/5	1 (1)	+ (2/2)	0/5	N/A	N/A
**Nephritis**	0/5	N/A	N/A	1/5	3 (3)	+ (1/1)	0/5	N/A	N/A
**Tracheitis/laryngitis**	1/5	2 (2)	+ (1/1)	1/5	2 (2)	+ (1/1)	0/5	N/A	N/A
**Myelitis (BM[b])**	1/5	1 (1)	+ (1/1)	2/5	2 (2)	+ (2/2)	0/5	N/A	N/A
**Pyogranulomas (BM[b])**	4/5	2.5 (2-4)	+ (4/4)	0/5	N/A	N/A	0/5	N/A	N/A
**Encephalitis**	0/5	N/A	N/A	0/5	N/A	N/A	1/5	3	+ (1/1)
**Lymphadenitis (MLN[c])**	4/5	3.2 (3-4)	+ (2/4)	0/5	N/A	N/A	0/5	N/A	N/A
**Lymphadenitis (TBLN[d])**	3/4[e]	2.6 (2-3)	+ (2/3)	0/5	N/A	N/A	0/5	N/A	N/A
**Lymphoid depletion (MLN[c])**	3/5	3.6 (3-4)	+ (2/3)	3/5	3.3 (3-4)	+ (3/3)	0/5	N/A	N/A
**Lymphoid depletion (TBLN[d])**	2/4	4 (4)	+ (1/2)	3/5	2.3 (2-3)	+ (3/3)	0/5	N/A	N/A
**Accumulation[f] (MLN[c])**	0/5	N/A	N/A	3/5	3.6 (3-5)	+ (3/3)	0/5	N/A	N/A
**Accumulation[f] (TBLN[d])**	0/5	N/A	N/A	4/5	2.25 (1-3)	+ (4/4)	0/5	N/A	N/A
**Necrosis[g] (MLN[c])**	0/5	N/A	N/A	1/5	3	+ (1/1)	0/5	N/A	N/A
**Necrosis[g] (TBLN[d])**	0/5	N/A	N/A	1/5	3	+ (1/1)	0/5	N/A	N/A

[a]Severity scores: 1: minimal, 2: mild, 3: moderate, 4: marked, 5: severe.

[b]Bone Marrow.

[c]Mediastinal lymph node.

[d]Tracheobronchial lymph node.

[e]One tracheobronchial lymph node was not examined.

[f]Expanded subcapsular sinus – with inflammatory cells, necrosis and fibrin.

[g]Necrosis within the cortex and medulla
